# Rationales for Prehabilitation Programs in Patients Preparing for Oncologic Surgery: A Systematic Review

**DOI:** 10.1245/s10434-025-18870-w

**Published:** 2026-01-09

**Authors:** Emine Akdemir, Wim G. Groen, Maike G. Sweegers, Bart C. Bongers, Anne M. May, Martijn M. Stuiver, Wim H. van Harten

**Affiliations:** 1 Division of Psychosocial Research and Epidemiology, The Netherlands Cancer Institute, Amsterdam, The Netherlands; 2 Center for Quality of Life, The Netherlands Cancer Institute, Amsterdam, The Netherlands; 3 Department of Medicine for Older People, Amsterdam UMC, Location Vrije Universiteit Amsterdam, Amsterdam, The Netherlands; 4 Amsterdam Movement Sciences, Ageing & Vitality, Rehabilitation & Development, Amsterdam, The Netherlands; 5 Amsterdam Public Health, Aging & Later Life, Amsterdam, The Netherlands; 6 Department of Nutrition and Movement Sciences, Institute of Nutrition and Translational Research in Metabolism (NUTRIM), Maastricht University, Maastricht, The Netherlands; 7 Department of Surgery, Institute of Nutrition and Translational Research in Metabolism (NUTRIM), Maastricht University, Maastricht, The Netherlands; 8 Julius Center for Health Sciences and Primary Care, University Medical Center Utrecht, Utrecht University, Utrecht, The Netherlands; 9 Division of Epidemiology and Data Science, Amsterdam UMC, location AMC, Amsterdam, the Netherlands; 10 Department of Health Technology and Services Research, University of Twente, Enschede, The Netherlands

**Keywords:** Oncological surgery, Physical exercise, Prehabilitation, Rationales, Systematic review

## Abstract

**Background:**

Prehabilitation aims to improve patients’ resilience to surgery and enhance postoperative recovery. Understanding rationales for prehabilitation content may identify opportunities for program optimization. This systematic review provides an overview of rationales, intervention, and outcomes used in prehabilitation studies in oncological populations.

**Patients and Methods:**

We searched the databases MEDLINE, Embase, and Scopus on 1 March 2024. Comparative prehabilitation studies including patients undergoing oncological surgery were included. Prehabilitation was defined as a preoperative exercise program, alone or combined with other components, with the explicit aim of improving postoperative outcomes. Extracted outcomes included reported rationales, program content, and primary endpoints.

**Results:**

In total, 140 studies (*N* = 24,925 patients) were included. Most (*N* = 125, 89%) reported a rationale for improving physical fitness, particularly cardiorespiratory fitness (*N* = 97, 69%). Psychological (*N* = 46, 33%) and metabolic (*N* = 28, 20%) rationales were reported less frequently. Rationales for specific attributes (e.g., intensity) were rarely described. Exercise was predominantly supervised (*N* = 57, 41%), and of these supervised sessions, most were prescribed three times per week (*N* = 25, 44%). Almost all studies (*N* = 121, 86%) included anaerobic exercise component at moderate-intensity continuous (*N* = 39, 32%) or high-intensity interval (*N* = 34, 28%) mode. Intended duration varied from 1 to 12 weeks. Most reported primary endpoints were surgical outcomes (e.g., complications) (*N* = 59, 42%), although definitions varied.

**Conclusions:**

Preoperatively improving physical fitness is a widely used rationale for prehabilitation; however, studies are implicit in rationales for specific program components. Content and duration of prehabilitation showed considerable variation, often determined by feasibility and time to surgery. Prehabilitation studies could benefit from standardized outcomes. Adopting a more mechanistically grounded approach could improve program design and possibly effectiveness.

**Trial Registration:**

The review was preregistered in International Prospective Register of Systematic Reviews (PROSPERO; CRD42024512892).

**Supplementary Information:**

The online version contains supplementary material available at 10.1245/s10434-025-18870-w.

The preoperative period is seen as a promising period for offering interventions to reduce surgical complications and accelerate and improve recovery. Such interventions are referred to as “prehabilitation,” which is defined as “a process in the continuum of care that occurs between the time of cancer diagnosis and the beginning of acute treatment.”^1^ Prehabilitation often includes a multimodal approach to enhance improvement in both physical and psychological resilience to surgery. Physical exercise training is often considered as a key component of this multimodal approach.

A prehabilitation program should ideally be aimed at the physiological conditions that are most relevant for recovery. Accordingly, the rationale for the content of the prehabilitation program should align with the underlying (psycho-)biological and physiological mechanisms through which the interventions are expected to work. For example, if the objective is to reduce pulmonary complications, implementing preoperative inspiratory muscle training (IMT) in combination with a strict smoking cessation program would target specific physical functions such as lung function, respiratory muscle strength, and/or improved clearance of secretions.^2^ Although generic programs may have generic benefits, it is likely that, for optimal prehabilitation, different intended outcomes require a different prehabilitation content.

The reporting on the content of prehabilitation programs is often poor,^3^ which hampers reproducibility of the intervention, limits comparison across studies, and challenges implementation into clinical practice. Moreover, poor reporting complicates the evaluation of the biological plausibility of the relationship between the intervention characteristics (e.g., exercise intensity) and intended outcome. To address this, intervention reporting guidelines such as the Template for Intervention Description and Replication (TIDieR)^4^ have been developed. This is specifically important for complex multimodal interventions, such as prehabilitation, where many modifiable variables, for example the frequency, intensity, type, and time (FITT) factors of exercise, influence its effectiveness. Regarding the intended outcome, if the goal is to reduce perioperative complications and accelerate and improve recovery, these clinical endpoints should also be a primary outcome in prehabilitation trials. Intermediate endpoints, such as improved preoperative physical fitness, are needed to understand whether the intervention works through the intended physiological pathway, but they are insufficient to demonstrate a clinical benefit.

The primary objective of this systematic review was to provide an overview of prehabilitation studies and identify the reported rationales for determining the content of prehabilitation programs for patients undergoing elective oncological surgery. Secondary objectives were to present the characteristics of the physical exercise training intervention and potential additional components and report on the choice of primary outcomes.

## Patients and Methods

We searched the databases MEDLINE, Embase, and Scopus on 1 March 2024. Keywords related to physical exercise training and prehabilitation comprised one group, while keywords pertaining to cancer types comprised the other. The complete search strategy is detailed in Appendix A. Additionally, a manual search of the reference lists of included studies and relevant review papers was conducted using a “backward snowballing” approach. Our systematic review is presented following the Preferred Reporting Items for Systematic Reviews and Meta-analysis (PRISMA) guidelines. The review was preregistered in International Prospective Register of Systematic Reviews (PROSPERO; CRD42024512892).

For the selection of studies, an eligible study should (1) be restricted to adult patients scheduled for elective oncological surgery; (2) investigate prehabilitation, defined as preoperative physical exercise training, with or without other components of prehabilitation with the explicit aim to improve postoperative outcomes; (3) include a control group; and (4) be written in the English or Dutch language. We excluded reviews, meta-analyses, and studies for which no full text was available. We defined physical exercise training as planned, structured and repetitive bodily movement to maintain or improve one or more components of physical fitness.^5^

### Study Selection, Data Extraction, and Data Synthesis

The titles and abstracts of all potentially eligible articles were screened by one reviewer (E.A.) using Rayyan systematic review software.^6^ If the title and abstract provided insufficient information, the full text was retrieved. A second reviewer (M.G.S.) independently verified a sample of eligible studies (10%). Discrepancies were resolved through discussion, and a third reviewer (M.M.S.) was consulted if needed.

One reviewer (E.A.) extracted all relevant information using a standardized data extraction form. The data extracted were general study information (first author, title, publication year, country, and study design), participant characteristics (cancer type and total sample size), high-risk patient inclusion criteria, rationale as reported by the authors, content of prehabilitation (unimodal or multimodal, duration, and delivery mode), details of physical exercise training (FITT factors and program duration), additional components (nutritional and psychosocial support), and the reported main outcome (definition and the timing of measurement). A second reviewer (W.G.) independently verified data extraction on a sample of included studies (10%). Discrepancies were resolved through discussion, and a third reviewer (M.M.S.) was consulted if needed.

The frequency of supervised and home-based exercise was summed and categorized as 1–2 times per week, 3 times per week, 3 times per week or more, and > 3 times per week. The intensity of the exercises was categorized into low, moderate, or high, following guidelines of the American College of Sports Medicine (ACSM)^7^ (Appendix B). For these categorizations, the components aerobic or interval and resistance exercises were evaluated separately. If the intensity of exercise was not clear, this information was extracted as “unknown.” The duration of exercise was categorized as < 60 min, 60 min, or > 60 min (all exercise types combined). All included types of exercise were presented (e.g., interval, resistance, and IMT). Additionally, the intended duration of the preoperative prehabilitation program was included.

To determine the rationale for the content of prehabilitation, potential mechanisms of prehabilitation effects on surgical complications were extracted as described by one narrative review.^8^ On the basis of that review, the following pathways were defined as potential mechanisms: (1) psychological; (2) physical; (3) physiological; (4) metabolic; (5) coagulation; (6) neuroendocrine; (7) cardiovascular; and (8) inflammatory. Given the primary interest in physical pathways, this category was further divided into subdomains:^9^ (a) cardiorespiratory fitness, (b) muscular strength, (c) muscular endurance, (d) flexibility or mobility, and (e) body composition. See Table 1 for an extended definition of these pathways. Since it was expected that rationales were reported relatively unstructured, all authors extracted this data on a sample of 5–10 papers each, and these were discussed in the authors’ group to reach agreement on mechanism scoring before data extraction of all papers by the lead author.

**Table 1:** Definition of pathways and examples extracted from the included prehabilitation studies

Pathway	Description	Example
Psychological	Mental resilience. Neuroprotection through stress regulation (e.g., cortisol modulation, enhanced neuroplasticity): reducing anxiety and depression were included as a pathway.	“*An observational study suggested that, compared with a historical control, a 4-week preoperative trimodal intervention comprising moderate intensity aerobic and resistance exercise, diet counseling with whey protein supplementation, and anxiety-reduction strategies was effective in improving preoperative functional walking capacity and accelerating postoperative recovery*.”^58^
Physical a. Cardiorespiratory fitness b. Muscular strength c. Muscular endurance d. Flexibility or mobility e. Body composition	Physical fitness and mobility. a. Improving cardiorespiratory fitness (terminology that meant cardiovascular fitness or endurance in context was also included [e.g., functional capacity]). b. Includes improving muscle strength and inspiratory and expiratory muscle training. c. Includes improving muscle endurance of inspiratory and expiratory muscles and also pelvic muscle training. d. Includes flexibility or mobility exercises. e. Includes improving body composition (e.g., muscle mass).	“*Conceptually there are three ways to limit the impact of colorectal cancer (CRC) surgery in elderly patients. First, through prehabilitation, by optimizing functional capacity prior to surgery using exercise training, nutritional support and optimizing comorbidity resulting in an increased resilience*.”^133^ “*However, because of the short time-window between decision for surgery and radical cystectomy, there is a need to optimize the exercise stimulus for cardiopulmonary adaptations, and the potential advantages of vigorous intensity interval exercise in this respect were recently highlighted. Interval training enables patients to undertake aerobic (endurance) exercise at a higher intensity than would be possible for continuous exercise at the same intensity but the feasibility of this exercise modality in bladder cancer patients awaiting radical cystectomy is unknown*.”^22^
Physiological	Body’s functional systems. Physiological consequences of physical exercise training: for example, cell proliferation for tissue repair and growth via growth factors.	“*Even a few sessions of HIIT appear sufficient to stimulate transcriptional mitochondrial biogenesis, inducing aerobic phenotypic changes characterized by increased mitochondrial expression/activity of proteins involved in fat oxidation, the tricarboxylic acid cycle, and the electron transport chain*.”^88^
Metabolic	Energy and nutrient regulation. Protein synthesis supporting muscle repair and metabolic health, including anabolic effect of protein.	“*Patients were instructed to ingest protein and/or the supplements within one hour of their exercise training to make use of the ‘anabolic window’, the moment at which muscle protein synthesis is the highest*”.^34^
Coagulation	Blood Clotting Balance. Anti-coagulation processes preventing excessive clotting.	“*Therefore, the preoperative respiratory rehabilitation program may demonstrate beneficial effects and improve the respiratory muscle strength. Preoperative respiratory rehabilitation, especially early mobilization, may improve the functional outcomes and cognitive and respiratory conditions, thereby reducing the risks of venous stasis and deep vein thrombosis*.”^156^
Neuroendocrine	The system through which the brain regulates hormone release to control physiological responses to stress.	
Cardiovascular	Circulatory health. Tissue oxygenation and vascularization ensuring optimal cardiovascular function.	“*Therefore, high-intensity interval training (HIIT) has been studied to strengthen aerobic abilities effectively. Traditionally, breath training is widely used to improve the inspiratory muscle endurance, exercise functions, and labored respiration of patients with COPD, wheezing, or lung dysfunction. Meanwhile, endurance training is preferentially adopted in rehabilitation to increase blood volume, cardiac output, and muscle oxygen extraction and thereby enhance exercise resistance and aerobic capabilities. In this study, a one-week HIIT combining both inspiratory training and endurance exercise was conducted to enhance the fitness, health status, and subsequent postoperative outcomes*.”^82^
Inflammatory	Immune system balance. Anti-inflammation to prevent tissue damage from chronic immune activation.	“*White blood cell and C-reactive protein have long been considered markers of inflammation. Recent studies have shown that postoperative CRP levels can predict the occurrence of incision infections and even intra-cavity infections. The value of prognosis is high, which can guide the prevention of perioperative complications. Therefore, white blood cell and C-reactive protein are used to reflect the effect of postoperative inflammation. Therefore, we believe that the appropriate preoperative pre-rehabilitation measures can effectively promote the recovery of postoperative gastrointestinal function in GC patients, reduce the physiological and psychological traumatic stress caused by surgery, and enable all aspects of patient function to reach the preoperative baseline level as soon as possible*.”^74^

The reported primary outcomes were classified on the basis of a categorization of a previous study:^10^ (1) patient-reported outcomes; (2) surgical outcomes; (3) physical or functional outcomes; (4) disease activity; (5) intervention delivery; and (6) cost-effectiveness.

## Results

**Fig. 1:**
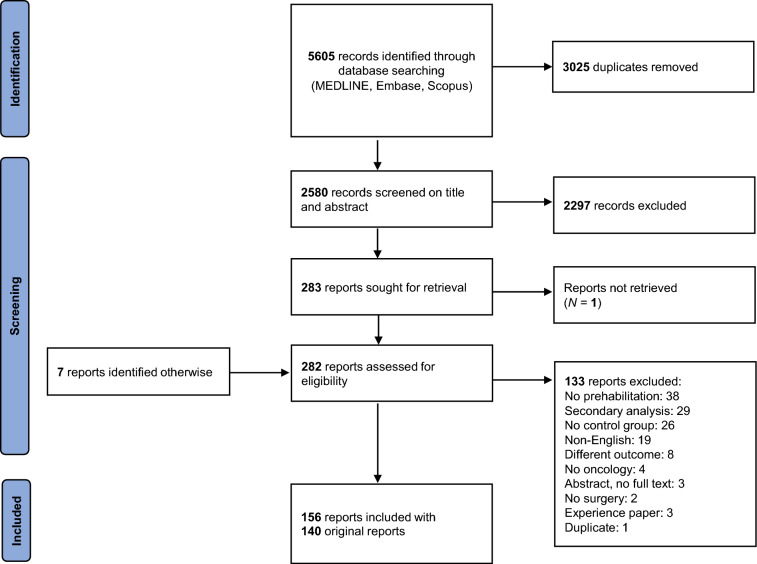
PRISMA flow chart

The literature search resulted in 5605 records—2580 records after removing duplicates. Of these, 2279 records were excluded on the basis of their title and abstract, leaving 283 full-text articles that were assessed for eligibility. Seven other studies were identified through snowballing. In total, 156 publications on 140 original studies were included. See Fig. 1 for the PRISMA flow chart.^11–166^

### Study Characteristics

We predominantly included randomized controlled trials (*N* = 53, 38%), study protocols (*N* = 33, 24%), and retrospective cohort studies (before–after study) (*N* = 21, 15%). Most studies were conducted in Canada (*N* = 19, 14%), the United Kingdom (*N* = 19, 14%), the Netherlands (*N* = 18, 13%), China (*N* = 15, 11%), or Spain (*N* = 13, 9%). Most studies included patients with colorectal cancer (*N* = 47, 34%), lung cancer (*N* = 32, 23%), or upper gastrointestinal cancer (*N* = 26, 19%). The median sample size across studies was 90 patients (interquartile range 50–157).

### General Prehabilitation Intervention Characteristics

In total, 70 studies (50%) implemented unimodal prehabilitation and 69 (49%) multimodal prehabilitation; 1 study evaluated both, in separate study arms. Among the multimodal interventions, other components were nutritional support (*N* = 66, 94%), psychosocial support (*N* = 47, 67%), and smoking cessation (*N* = 21, 30%) and components such as medical optimization, education, sleep optimization, and bowel decompression (*N* = 17, 24%).

### Reported Rationales

**Fig. 2:**
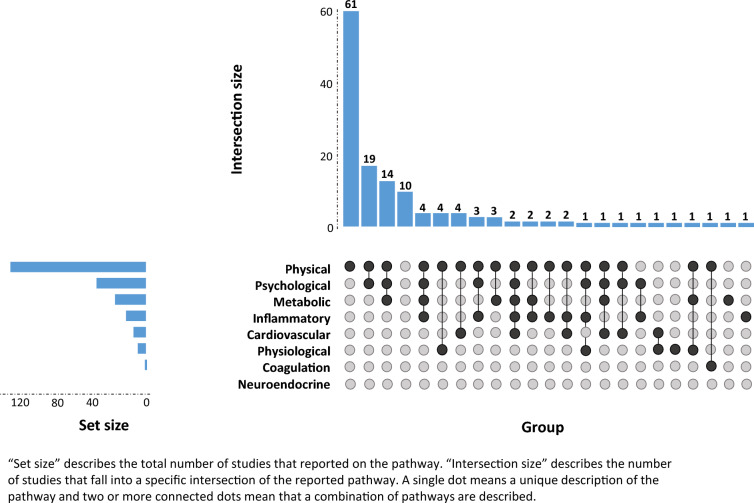
UpSet plot of the reported pathways for the rationales in the included prehabilitation studies

The majority of the included studies (*N* = 125, 89%) hypothesized the intervention would act through a physical pathway. Specifically, studies targeted cardiorespiratory fitness (*N* = 97, 69%), muscular strength (*N* = 59, 42%), muscular endurance (*N* = 3, 2%), flexibility or mobility (*N* = 5, 4%), and body composition (*N* = 37, 26%). The psychological pathway was also commonly reported on (*N* = 46, 33%), followed by the metabolic pathway (*N* = 28, 20%), the inflammatory pathway (*N* = 18, 13%), the cardiovascular pathway (*N* = 11, 8%), the physiological pathway (*N* = 8, 6%), and the coagulation pathway (*N* = 1, 1%). No studies explicitly referred to a neuroendocrine pathway. Ten studies did not report any specific rationale (7%). Table 1 provides an example for each pathway, and Fig. 2 visualizes the distributions of reported pathways and their combinations for the rationale using an UpSet plot.

### Prehabilitation: Physical Exercise Training

The physical exercise training part of the prehabilitation programs was delivered in supervised settings (*N* = 57, 41%), unsupervised home-based settings (*N* = 46, 33%), or a combination of both (*N* = 27, 19%). In five studies, supervised or home-based exercise was either determined by patient preference or tailored according to the patient’s physical condition. In five studies the delivery mode was unclear.

Within the 57 supervised exercise studies the most used exercise frequency was 3 times per week (*N* = 25, 44%). In contrast, for the 46 home-based and 27 combined supervised and home-based exercise studies, the most used frequency was > 3 times per week (*N* = 31, 67%, *N* = 23, 85%).

**Fig. 3:**
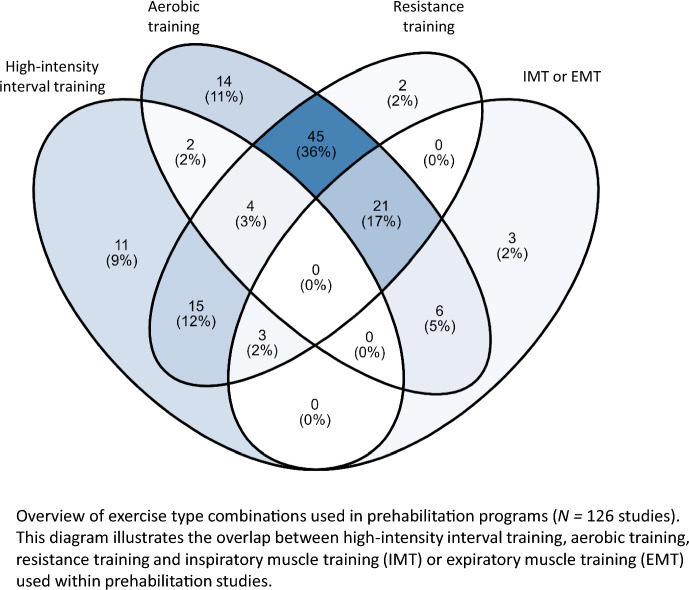
Venn diagram of physical exercise training combinations in prehabilitation studies

Almost all studies (*N* = 121, 86%) included an aerobic exercise component. The reported exercise intensities were high-intensity (interval training) (*N* = 34, 28%), high-intensity (continuous) (*N* = 7, 6%), moderate-intensity (*N* = 39, 32%), and low-intensity (*N* = 22, 18%), while 25 studies (18%) did not specify the intensity. In six studies, an additional aerobic component was included in the home-based part of the physical exercise training program, alongside high-intensity interval training. Most studies (*N* = 90, 64%) also included a resistance training component, either at moderate intensity (*N* = 33, 36%) or high-intensity (*N* = 5, 6%). In 52 studies (58%), the resistance training intensity was not reported. In total there, were 33 studies that had IMT or expiratory muscle training (EMT) as the type of exercise. Figure 3 presents a Venn diagram of 126 studies that used one or more of the following exercise types: aerobic training, high-intensity interval training, resistance training, or IMT or EMT. The most common combination was aerobic and resistance exercises. Five studies included pelvic muscle exercises (one study within hatha yoga and one alongside aerobic and resistance exercise training), one study only included shoulder exercises, one study did only breathing exercises, one study did breathing exercises and stretching, and seven studies did not report the type of exercise.

Program duration was flexible and depended mostly on the date of surgery. The intended duration varied from 1 week to a maximum duration of 12 weeks.

### Prehabilitation: Nutritional and Psychosocial Support

Of the 66 studies that included nutritional support, most had a combination of nutritional counseling and supplementation (*N* = 35, 53%). Supplementation, often whey protein, was often linked to the anabolic effects on muscle mass in combination with physical exercise training. Of the 47 studies that included psychological support, most provided counseling with strategies related to anxiety reduction (*N* = 36, 77%), mindfulness (*N* = 5, 10%), education (*N* = 2, 4%), cognitive behavioral therapy (*N* = 1, 2%), behavior change techniques (*N* = 1, 2%), and breathing relaxation and personalized music therapy and assisted sleep (*N* = 2, 4%).

### Reported Outcomes

The majority of studies had surgical outcome as primary endpoint (*N* = 59, 42%). The second-most reported primary endpoints were physical or functional outcomes (*N* = 54, 39%). Several studies had intervention delivery (feasibility) as primary endpoint (*N* = 21, 15%). Two studies had more than one primary endpoint. One study had a combination of physical or functional outcome, a surgical outcome, and intervention delivery outcome, and one study had a physical or functional and surgical outcome (*N* = 2, 2%). Only two studies (1%) assessed cost-effectiveness, and two (1%) focused on patient-reported or patient-related outcomes.

**Fig. 4:**
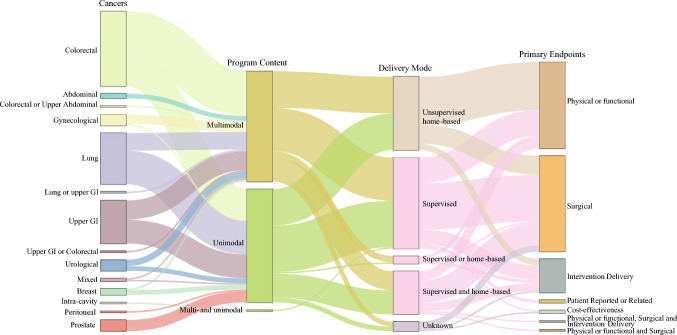
Sankey plot depicting prehabilitation studies going from oncological groups, to program content, delivery mode, and primary endpoints. The width of the flows is proportional to the number of studies. *GI* gastrointestinal

Figure 4 provides a graphic overview of prehabilitation studies among oncological tumor groups, the delivery setting, program content, and primary outcomes. Details of the prehabilitation intervention, the reported rationales, and reported outcomes are presented in Appendix C.

### High-Risk Patient Inclusion Criteria

A total of 40 studies (29%) applied a “high-risk status” of patients as an inclusion criterion for prehabilitation. Single or combined risk factors defining this status were age, frailty, pulmonary function, comorbidities, performance status, American Society of Anesthesiologists classification, physical fitness and activity, tumor related characteristics, and blood values. Definitions and thresholds for these risk factors varied across studies and are summarized in Table 2.

**Table 2:** High-risk patient inclusion criteria applied in prehabilitation studies

Risk factor	Tool or definition and references to the specific studies in which it was applied
Age	Age
	≥ 60 years old^48^
	≥ 65 years old^16,45,65,150^
	≥ 70 years old^41,62,68,71,78,146^
	≥ 75 years old^81,97,127,133^
Frailty	Fried Frailty Index > 1^37^; ≥ 2^98^; ≥ 4^99,100^
	G8 screening tool for older people ≤ 14^39^; ≤ 14/17^139^
	Edmonton Frail Scale > 5^56^; ≥ 6^116^
	Clinical Frailty Score > 4^148^
Pulmonary function	Predicted postoperative FEV_1_ and/or predicted postoperative lung diffusion capacity for carbon monoxide < 60%^23^
	Bronchial hyperresponsiveness and PEF < 250 L/min/kg, 4.1.0 L < FEV_1_ < 1.2 L and 40% < FEV_1_% < 60%^57^
	FEV_1_/FVC ratio ≤ 70% or prior history of thoracic surgery^68^
	ppoFEV_1_% < 60% or ppoDLCO < 60%^81^
	ppoFEV_1_% < 60%^82^
	FEV_1_ < 1000 mL^122^
	FEV_1_ ≤ 80% of predicted value^127^
	FEV_1_ ≤ 60%^157^
Comorbidities	COPD^26,68,84,122,128,134,157^
	BMI < 18.5 kg/m^2^ ^32^; ≥ 28 kg/m^2^ ^157^; > 30 kg/m^2^ ^68,81,127,140^
	Smoking status (smoking history)^57,81,140,157^
	Weight loss (around 10–15% in the last 6 months)^32,103^
	Malnutrition risk (ESPEN) or > 2 alcoholic drinks per day (1 drink = 12 g ethanol)^140^
	Mental impairment (HADS anxiety > 6 and/or HADS depression > 4)^103^
	Metabolic syndrome^42,43^
	Two or more comorbidities^127^
Performance status	WHO I–II^31^
ASA classification	ASA score III–VI^23,45,62^
Physical fitness and activity	Maximal walking speed < 2 m/s^16^
	VO_2_ at the VAT < 11 mL/kg/min^27,28^
	VO_2_max ≤ 20 mL/kg/min^33^
	6MWT distance < 400 m or DASI < 46^103^
	Physical inactivity (defined as < 30 minutes per day or < 3.5 hours per week)^140^
	Timed up-and-go test ≥ 15 seconds^32^
Tumor-related characteristics	Right-sided obstructing colon^30^
Blood markers	Anemia (hemoglobin > 7 mmol/L; albumin <30 g/L)^32^

*6MWT* 6-minute walk test, *ASA* American Society of Anesthesiologists, *BMI* body mass index, *ESPEN* The European Society for Clinical Nutrition and Metabolism, *COPD* chronic obstructive pulmonary disease, *DASI* Duke Activity Status Index, *FEV*_*1*_ forced expiratory volume in the first second, *FVC* forced vital capacity, *G8* Geriatric 8, *HADS* hospital anxiety and depression scale, *PEF* peak expiratory flow, *ppoDLCO* postoperative predicted diffusing capacity of the lungs for carbon monoxide, *ppoFEV*_*1*_*%* postoperative predicted percentage of forced expiratory volume in the first second, *VAT* ventilatory anaerobic threshold, *VO*_*2*_ oxygen uptake, *VO*_*2*_*max* maximal oxygen uptake, *WHO* World Health Organization

Most studies included only adult patients (*N* = 94, 67%; with one study including patients aged ≥ 16 years). Some studies included patients above a certain age or within a specific age range, but without explicitly referring to age as a risk factor (*N* = 25, 18%), while a few included patients younger than < 65 years or within 65–74 years of age, < 70 years of age, or < 75 years of age (*N* = 4, 3%). Conversely, age was explicitly considered as a risk factor in 15 studies (10%).

## Discussion

This review provides an overview of prehabilitation studies in patients undergoing major oncological surgery, with a primary focus on the reporting of hypothesized mechanistic rationales for the content of the prehabilitation programs, as well as on the alignment of program content with those rationales. It was found that most studies explicitly targeted physical pathways, focusing on improving outcomes through improved cardiorespiratory fitness and muscular strength. The rationale was often described unsystematically and in rather general terms, while terminology for seemingly similar concepts varied, for example, “improving functional capacity” or “enhancing physical fitness.” Notably, almost one in three studies explicitly included a “high-risk” population, albeit based on varying nonstandardized criteria. Interestingly, some studies used (high) age as an inclusion criterion without explicitly categorizing it as a high-risk variable.

Prehabilitation, and specifically preoperative physical exercise training, has the potential to exert effects through multiple interrelated (psycho)biological and physiological pathways. These were however seldom explicitly described as being related to the nature and specific characteristics of interventions. Physical exercise training is a potent physiological stimulus, with acute effects that could be relevant in the preoperative period. Even a single bout of aerobic or resistance exercise can acutely increase blood flow, enhance oxygen delivery to tissues,^167^ improve insulin sensitivity, promote metabolic flexibility,^168^ and modulate inflammatory cytokines.^169^ Repeated sessions can induce adaptations in cardiovascular health,^170^ in skeletal muscle strength,^171^ and on the neuroendocrine system.^172^ Moreover, physical exercise training can positively influence psychological resilience,^173^ reduce fatigue,^174^ and improve sleep and physical function,^175^ all of which may contribute to better postoperative recovery. However, specificity is a key principle of exercise physiology; different prescriptions will elicit different adaptations.

There are two key considerations when designing a prehabilitation program. First, the ambition to achieve specific outcomes through hypothesized mechanistic pathways, and second, the feasibility constraints related to patient acceptance and the available time before surgery. In this review, it was found that in general the frequency of physical exercise was 3 times per week in the supervised setting, but the intensity, type of exercise, duration, and delivery mode varied within studies, mostly without a clear link between design and presumed mechanism. These variations seemed related to pragmatic reasons or personal experience with previous exercise interventions. As positive exceptions, Banerjee et al.^22^ and van Rooijen et al.^147^ explained that their choice for high-intensity interval training was made on the basis of the short window of opportunity, on the one hand, and what is needed to achieve the cardiopulmonary improvements required to impact postoperative outcomes, on the other hand.

The duration of prehabilitation was often determined by the time available before surgery, rather than based on what would be needed to achieve a certain outcome. The duration is one of the main challenges in prehabilitation, as the current quality paradigm in oncology care is heavily driven by the notion that faster surgery equals better care. However, there is evidence that delaying surgery does not lead to poorer outcomes in colon cancer care,^176^ whereas prehabilitation improves such outcomes.^107^ This suggests that it could be acceptable—if not preferable—to promote a minimum length of the preoperative period in treatment guidelines to enable prehabilitation, unless there is evidence that this is unsafe.

We observed variations in outcome definitions and the timing of outcome measurements. Despite targeting similar endpoints, studies varied widely in how outcomes such as surgical complications (e.g., Clavien–Dindo, comprehensive complication index on 30 versus 90 days following surgery) or physical fitness (e.g., 6-minute walk test distance, maximal oxygen uptake, oxygen uptake at the ventilatory anaerobic threshold) were defined and measured, which can complicate comparability and underscores the need for standardized outcomes that are aligned with the underlying rationale. These variations and the need for core outcome sets for prehabilitation has been previously recognized,^177^ and Rammant et al. are currently working on a core outcome set for prehabilitation.^178^

This review focused on the preoperative period. However, developments in cancer care have led to complex and varying treatment trajectories, depending on cancer type, stage, and therapeutic aim. Courneya et al.^179^ described this complexity, highlighting the wide range of interventions that can be implemented, including those during neoadjuvant treatment before surgery. Although the aim of these interventions may vary, most prehabilitation studies typically focus on improving surgical outcomes or physical fitness, rather than oncological outcomes. This means that the intervention should align with the treatment phase. For example, in the ENHANCE trial,^11^ patients who are included during neoadjuvant treatment receive structured aerobic and resistance exercise twice weekly, with the aim to *maintain* physical fitness despite treatment toxicity. In contrast, during the preoperative period following neoadjuvant treatment, patients receive a structured program with interval, resistance, and relaxation exercises 3 times per week, with the aim to *improve* physical fitness prior to surgery. Some studies focus on oncological outcomes during neoadjuvant treatment, such as NK-cell infiltration in patients with prostate cancer,^180^ KI-67 proliferation in patients with breast cancer,^181^ time from neoadjuvant treatment to surgery,^182^ and chemotherapy response^183^ in esophageal cancer. These examples illustrate the diversity in aims and the need to distinguish between interventions implemented during other treatment trajectories and those specifically in the preoperative period.

The major strength of this review is the inclusion of a large range of prehabilitation studies in the oncological population. Since the rationales of the included studies were often implicit, or were stated scattered throughout the publication and supplementary documents, the extraction of this variable was however subject to interpretation. This underscores once more that future prehabilitation studies would benefit from a more explicit and structured reporting of intervention rationale to align intervention and outcomes.

### Future Directions

The current definition of prehabilitation is broad, and we found heterogeneous prehabilitation programs in terms of content, inclusion criteria, duration, delivery, and outcomes. To advance prehabilitation care, it is important to generate guidance on treatment “window” when prehabilitation should be implemented, and to explicitly align the aim and content of the program with the outcomes. Clarity is needed to distinguish prehabilitation interventions from other perioperative care. For instance, medical optimization and smoking cessation are inconsistently classified as part of either prehabilitation or preoperative optimization, while they can also be considered standard of care. When defining inclusion criteria for prehabilitation, there is a need to better differentiate between subgroups, as many studies do not apply risk stratification. Moreover, there is currently no clear or consistent definition of what constitutes a “high-risk” population, as criteria vary across studies. Although it seems intuitive and clinically logical to prioritize prehabilitation for those most at risk of adverse outcomes, limited studies have directly compared the effectiveness of prehabilitation between high-risk and lower-risk patients. Future research should include post hoc analyses of existing trial data or use prediction modeling to identify criteria for selecting high-risk patients. Selecting the right patients is especially relevant in healthcare systems where reimbursement decisions depend on evidence from prehabilitation effectiveness trials, such as the National Health Care Institute in the Netherlands.

When designing physical exercise interventions, the FITT factors and the available duration in the preoperative window must be considered when designing the intervention to elicit maximum effect. Prehabilitation trials still lack consistency on reporting intervention details (e.g., prescribed FITT factors and patient adherence), as has been previously addressed.^3^ To ensure transparency and reproducibility, reporting guidelines such as the TIDieR or International Consensus on Therapeutic Exercise and Training (i-CONTENT) checklist should be used. Apart from a well-described content based on a rationale, delivering effective physical exercise training in cancer care demands adaptive, patient-centered prescriptions with dynamic monitoring to ensure feasibility, engagement, and lasting impact in real-world settings.^184^

## Conclusions

Despite the intertwined (psycho-)biological and physiological pathways that are involved in prehabilitation, few studies in this review explicitly linked these to their intervention rationale and many focused solely on the physical pathway. Explicit and detailed alignment of the rationale and content was often lacking. Adopting a more mechanistically grounded approach to prehabilitation could improve intervention design and possibly effectiveness.

## Supplementary Information

Below is the link to the electronic supplementary material.

Supplementary file1 (DOCX 76 KB)

Supplementary file2 (DOCX 268 KB)

## Data Availability

The datasets used and/or analyzed in the current review are available from the corresponding author on reasonable request.
