# Supplementary material for: Rationales for Prehabilitation Programs in Patients Preparing for Oncologic Surgery: A Systematic Review
Source: Ann Surg Oncol. 2026 Jan 9;33(3):2414–30. doi: 10.1245/s10434-025-18870-w (PMC12901104; doi:10.1245/s10434-025-18870-w)
Supplement: Supplementary file 1 — Supplementary file1 (DOCX 76 KB) [file 10434_2025_18870_MOESM1_ESM.docx]

**Appendix A**

## Definitive search 1-3-2024

### Medline

(exp Preoperative Exercise/ or (prehab* or pre-hab* or ((pre-op* or preop* or peri-op* or periop* or presurg* or pre-surg*) adj3 rehab*)).ti,ab,kf. or (exp Preoperative Period/ and exp Exercise/) or ((pre-op* or preop* or peri-op* or periop* or presurg* or pre-surg*) adj4 (exercis* or "physical activit*" or "physical therap*" or "physiotherap*" or "physical education*" or "muscle training*" or "interval training*" or weightlift* or "weight lift*" or pilates or cycling or aerobic* or movement or yoga or walk* or sport* or lifestyle or "life style")).ti,ab,kf.) and (exp Neoplasms/ or (adenocarcinom* or adenoma* or blastoma* or cancer* or carcinom* or carcinosarcoma* or cholangiocarcinom* or chordoma* or germinoma* or gonadoblastoma* or hepatoblastoma* or "hodgkin disease" or "hodgkin's disease" or "hodgkins disease" or leukaemi* or leukemi* or lymphangioma* or lymphangiomyoma* or lymphangiosarcoma* or lymphom* or malignan* or melanom* or meningioma* or mesenchymoma* or mesonephroma* or metasta* or myeloma or neoplas* or neuroma* or nsclc or oncogen* or oncolog* or paraneoplastic or plasmacytoma* or sarcoma* or teratocarcinoma* or teratoma* or tumor* or tumour*).ti,ab,kf.)

### Embase.com

('preoperative exercise'/exp OR (prehab* OR pre-hab* OR ((pre-op* OR preop* OR peri-op* OR periop* OR presurg* OR pre-surg* ) NEAR/3 rehab* )):ti,ab,kw OR ('preoperative period'/exp AND exercise/exp) OR ((pre-op* OR preop* OR peri-op* OR periop* OR presurg* OR pre-surg* ) NEAR/4 (exercis* OR 'physical activit*' OR 'physical therap*' OR physiotherap* OR 'physical education*' OR 'muscle training*' OR 'interval training*' OR weightlift* OR 'weight lift*' OR pilates OR cycling OR aerobic* OR movement OR yoga OR walk* OR sport* OR lifestyle OR 'life style')):ti,ab,kw) AND (neoplasm/exp OR (adenocarcinom* OR adenoma* OR blastoma* OR cancer* OR carcinom* OR carcinosarcoma* OR cholangiocarcinom* OR chordoma* OR germinoma* OR gonadoblastoma* OR hepatoblastoma* OR 'hodgkin disease' OR 'hodgkin s disease' OR 'hodgkins disease' OR leukaemi* OR leukemi* OR lymphangioma* OR lymphangiomyoma* OR lymphangiosarcoma* OR lymphom* OR malignan* OR melanom* OR meningioma* OR mesenchymoma* OR mesonephroma* OR metasta* OR myeloma OR neoplas* OR neuroma* OR nsclc OR oncogen* OR oncolog* OR paraneoplastic OR plasmacytoma* OR sarcoma* OR teratocarcinoma* OR teratoma* OR tumor* OR tumour* ):ti,ab,kw)

### Scopus

(TITLE-ABS-KEY(prehab* OR pre-hab* OR ((pre-op* OR preop* OR peri-op* OR periop* OR presurg* OR pre-surg* ) W/3 rehab* )) OR TITLE-ABS-KEY((pre-op* OR preop* OR peri-op* OR periop* OR presurg* OR pre-surg* ) W/4 (exercis* OR "physical activit*" OR "physical therap*" OR physiotherap* OR "physical education*" OR "muscle training*" OR "interval training*" OR weightlift* OR "weight lift*" OR pilates OR cycling OR aerobic* OR movement OR yoga OR walk* OR sport* OR lifestyle OR "life style" ))) AND (TITLE-ABS-KEY(adenocarcinom* OR adenoma* OR blastoma* OR cancer* OR carcinom* OR carcinosarcoma* OR cholangiocarcinom* OR chordoma* OR germinoma* OR gonadoblastoma* OR hepatoblastoma* OR "hodgkin disease" OR "hodgkin's disease" OR "hodgkins disease" OR leukaemi* OR leukemi* OR lymphangioma* OR lymphangiomyoma* OR lymphangiosarcoma* OR lymphom* OR malignan* OR melanom* OR meningioma* OR mesenchymoma* OR mesonephroma* OR metasta* OR myeloma OR neoplas* OR neuroma* OR nsclc OR oncogen* OR oncolog* OR paraneoplastic OR plasmacytoma* OR sarcoma* OR teratocarcinoma* OR teratoma* OR tumor* OR tumour ))

**Appendix B**

Exercise intensity based on American College of Sports Medicine (ACSM) guidelines (8)

| **Aerobic exercise** | | | |
| --- | --- | --- | --- |
|  | HRmax (%) | HRR (%); VO_2_max (%); VO_2_r (%) | RPE |
| Low | <64 | <40 | <11 |
| Moderate | 64 - <77 | 40 - <60 | 12 - 13 |
| High | 77 - 100 | 60 - 100 | >14 |
| **Resistance exercise** | | | |
| Low | Less than 8 repetitions at an intensity at or below 40% to 80% of a 1-RM | | |
| Moderate | 8 to 12 repetitions at an intensity of 40% to 80% of a 1-RM | | |
| High | More than 12 repetitions at an intensity at or above 40% to 80% of a 1-RM | | |

HRmax = maximal heart rate, HRR = heart rate reserve, VO_2_max = maximal oxygen uptake, VO_2_r = oxygen uptake reserve, RPE = rating of perceived exertion, 1-RM = one repetition maximum

**Appendix C**

**Details prehabilitation intervention, rationales, and outcomes**

1. **Colorectal Cancer**

| **Author and title** | **Study info** | **Delivery** | **Timing** | **Other components** | **Main outcome** | **Pathways** | **FITT and duration** |
| --- | --- | --- | --- | --- | --- | --- | --- |
| Amaro-Gahete et al.; Multidisciplinary Prehabilitation and Postoperative Rehabilitation for Avoiding Complications in Patients Undergoing Resection of Colon Cancer: Rationale, Design, and Methodology of the ONCOFIT Study | 2022 \| Spain \| RCT (protocol) \| SS: 90 \| RS: No | Supervised | Preoperatively and postoperatively | Nutritional support; Psychosocial support | Surgical outcomes; CCI; 90 days postoperatively, 1 year postoperatively | Psychological; Physical (A, B, E); Inflammatory | F: 3x/week;  I: High, High;  T: 60 min;  T: Interval, Resistance;  D: 4 weeks |
| Andersson et al.; CANOPTIPHYS study protocol: Optimising PHYSical function before CANcer surgery: effects of pre-operative optimisation on complications and physical function after Gastrointestinal cancer surgery in older people at risk-a multicentre, randomised, parallel-group study | 2023 \| Sweden \| RCT (protocol) \| SS: 160 \| RS: Yes | Supervised and home-based | Preoperatively |  | Physical or functional outcomes, Surgical outcomes; 6MWD, Clavien-Dindo; Baseline and before surgery, 30 days postoperatively | Physical (B) | F: >3x/week;  I: Moderate, Moderate;  T: Unknown;  T: Aerobic, Resistance, IMT;  D: 2 weeks |
| PREPARE-ABC; SupPoRtive Exercise Programmes for Accelerating REcovery after major abdominal Cancer surgery trial (PREPARE-ABC): Study protocol for a multicentre randomized controlled trial | 2021 \| UK \| RCT (protocol) \| SS: 1146 \| RS: No | Supervised and home-based | Preoperatively and postoperatively |  | Surgical outcomes; Clavien-Dindo; 30 days postoperatively | Physical (A, E); Cardiovascular | F: >3x/week;  I: Moderate, High, Unknown;  T: <60 min;  T: Aerobic, Interval, Resistance;  D: 3 - 4 weeks |
| Anthuber et al.; Influence of perioperative step volume on complication rate and length of hospital stay after colorectal cancer surgery (IPOS trial): study protocol for a randomised controlled single-centre trial at a German university hospital | 2023 \| Germany \| RCT (protocol) \| SS: 222 \| RS: No | Home-based | Preoperatively |  | Surgical outcomes; LOS; Hospitalization period | Physical (No) | F: >3x/week;  I: Moderate;  T: Unknown;  T: Aerobic;  D: 1 - 6 weeks |
| Berkel et al.; The effects of prehabilitation versus usual care to reduce postoperative complications in high-risk patients with colorectal cancer or dysplasia scheduled for elective colorectal resection: study protocol of a randomized controlled trial* | 2018 \| The Netherlands \| RCT (protocol) \| SS: 86 \| RS: Yes | Supervised | Preoperatively |  | Surgical outcomes; Clavien-Dindo; 30 days postoperatively | Physical (A, B) | F: 3x/week;  I: High, Moderate;  T: 60 min;  T: Interval, Resistance;  D: 3 weeks |
| Boeding et al.; Postponing surgery to optimise patients with acute right-sided obstructing colon cancer - A pilot study | 2023 \| The Netherlands \| Retrospective cohort \| SS: 54 \| RS: Yes | Unknown | Preoperatively | Nutritional support; Bowel decompression | Intervention Delivery; Feasibility and safety of medical optimization (determined by the treating physician); Intervention period | Physical (No) | F: Unknown;  I: Unknown;  T: Unknown;  T: Unknown;  D: Unspecified |
| Bojesen et al.; Preoperative multimodal prehabilitation before elective colorectal cancer surgery in patients with WHO performance status I or II: randomized clinical trial | 2023 \| Denmark \| RCT \| SS: 36 \| RS: Yes | Supervised | Preoperatively | Nutritional support; Medical optimization | Patient Reported/Related Outcomes; Postoperative recovery assessed via Quality of Recovery 15 questions; 3 days postoperatively | Physical (A) | F: 3x/week;  I: High, Unknown;  T: Unknown;  T: Interval, Resistance;  D: 4 weeks |
| Bojesen et al.; Effect of modifying high-risk factors and prehabilitation on the outcomes of colorectal cancer surgery: controlled before and after study | 2022 \| Denmark \| Unrandomized controlled study \| SS: 1591 \| RS: Yes | Supervised | Preoperatively | Nutritional support; Medical optimization | Surgical outcomes; Composite endpoint: LOS more than 10 days, unplanned admission to intensive care, readmission within 30 days, a complication with a Clavien-Dindo score of 3a or higher within 30 days, or death within 30 days after surgery; 30 days postoperatively | Physical (A) | F: Unknown;  I: Unknown;  T: Unknown;  T: Unknown;  D: 4 weeks |
| Bousquet-Dion et al.; Evaluation of supervised multimodal prehabilitation programme in cancer patients undergoing colorectal resection: a randomized control trial | 2018 \| Canada \| RCT \| SS: 63 \| RS: No | Supervised and home-based | Preoperatively | Nutritional support; Psychosocial support | Physical or functional outcomes; 6MWD; Baseline, before surgery, 4 and 8 weeks postoperatively | Psychological; Physical (A); Metabolic | F: >3x/week;  I: Unknown, Unknown;  T: 60 min;  T: Aerobic, Resistance;  D: 4 weeks |
| Boyle et al.; Multimodal prehabilitation service for patients with colorectal cancer: the challenges of implementation | 2023 \| UK \| Cohort study \| SS: 49 \| RS: No | Home-based | Preoperatively | Nutritional support; Psychosocial support | Intervention Delivery; Feasibility, not specified; Intervention period | Psychological; Physical (A) | F: 3x/week;  I: Moderate, Unknown;  T: Unknown;  T: Aerobic, Resistance, IMT;  D: Unspecified |
| Carli et al.; Effect of Multimodal Prehabilitation vs Postoperative Rehabilitation on 30-Day Postoperative Complications for Frail Patients Undergoing Resection of Colorectal Cancer: A Randomized Clinical Trial | 2020 \| Canada \| RCT \| SS: 110 \| RS: Yes | Supervised and home-based | Preoperatively | Nutritional support; Psychosocial support; Smoking cessation | Surgical outcomes; CCI; 30 days postoperatively | Psychological; Physical (A, B, E); Metabolic | F: >3x/week;  I: Moderate, Unknown;  T: 60 min;  T: Aerobic, Resistance, Stretching;  D: 4 weeks |
| Cerdan Santacruz et al.; "Optimal recovery" after colon cancer surgery in the elderly, a comparative cohort study: Conventiol care vs. enhanced recovery vs. prehabilitation | 2023 \| Spain \| Cohort study \| SS: 153 \| RS: Yes | Unknown | Preoperatively | Nutritional support; Psychosocial support | Surgical outcomes; Postoperative course without major complications, no mortality, hospital discharge before the fifth postoperative day and without readmission; 30 days postoperatively |  | F: 3x/week or more;  I: Unknown, Unknown;  T: Unknown;  T: Aerobic, Resistance, IMT;  D: 4 weeks |
| de Klerk et al.; A multimodal prehabilitation program in high-risk patients undergoing elective resection for colorectal cancer: A retrospective cohort study | 2021 \| The Netherlands \| Retrospective cohort \| SS: 351 \| RS: Yes | Supervised and home-based | Preoperatively | Nutritional support; Smoking cessation; Medical optimization | Surgical outcomes; Proportion of postoperative complications; 90 days postoperatively | Physical (A, E); Metabolic | F: >3x/week;  I: Low (not specified), High;  T: Unknown;  T: Aerobic, Interval;  D: 4 weeks |
| Dronkers et al.; Preoperative therapeutic programme for elderly patients scheduled for elective abdominal oncological surgery: a randomized controlled pilot study | 2010 \| The Netherlands \| RCT \| SS: 42 \| RS: Yes | Supervised and home-based | Preoperatively |  | Intervention Delivery, Physical or functional outcomes, Surgical Outcomes; Feasibility assessed via compliance and AE, inspiratory muscle endurance (MicroRMA), postoperative complications and LOS; Intervention period and postoperatively | Physical (A, B) | F: >3x/week;  I: Moderate, High;  T: 60 min;  T: Aerobic, Resistance, IMT;  D: 2 - 4 weeks |
| Dunne et al.; Randomized clinical trial of prehabilitation before planned liver resection | 2016 \| UK \| RCT \| SS: 38 \| RS: No | Supervised | Preoperatively |  | Physical or functional outcomes; anaerobic threshold using CPET; Baseline, before surgery | Psychological; Physical (A, E); Metabolic | F: 3x/week;  I: High;  T: <60 min;  T: Interval;  D: 4 weeks |
| Estrada et al.; Comparative study using propensity score matching alysis in patients undergoing surgery for colorectal cancer with or without multimodal prehabilitation | 2023 \| Brazil \| Unrandomized controlled study \| SS: 210 \| RS: No | Supervised | Preoperatively | Nutritional support; Psychosocial support | Surgical outcomes; LOS, postoperative complication rate, postoperative emergency room visit, and hospital readmission; Postoperatively | Psychological; Physical (A, E) | F: 3x/week;  I: High;  T: <60 min;  T: Aerobic;  D: 4 weeks |
| Fabulas et al.; Pre-habilitation before colorectal cancer surgery could improve postoperative Gastrointestinal function recovery: a case-matched study | 2022 \| France \| Unrandomized controlled study \| SS: 113 \| RS: No | Supervised | Preoperatively | Nutritional support; Psychosocial support; Occupational therapy | Surgical outcomes; Gastrointestinal motility recovery defined as tolerance to solid food and flatus and/or stools; Surgery date and day of recovery | Physical (A, B, E); Metabolic; Inflammatory | F: Unknown;  I: Unknown;  T: Unknown;  T: Unknown;  D: 2 - 7 weeks |
| Furyk et al.; Prehabilitation for Frail Patients Undergoing Colorectal Surgery: Lessons Learnt From a Randomised Feasibility Study | 2021 \| Australia \| RCT \| SS: 5 \| RS: Yes | Supervised | Preoperatively | Nutritional support | Physical or functional outcomes; 6MWD; Baseline, 30 days postoperatively | Physical (A, B) | F: 3x/week;  I: Unknown, Moderate;  T: 60 min;  T: Interval, Resistance, Balance;  D: 4 weeks |
| Gillis et al.; Prehabilitation versus rehabilitation: a randomized control trial in patients undergoing colorectal resection for cancer | 2014 \| Canada \| RCT \| SS: 77 \| RS: No | Home-based | Preoperatively | Nutritional support; Psychosocial support | Physical or functional outcomes; 6MWD; Baseline, 8 weeks postoperatively | Psychological; Physical (A); Metabolic; Inflammatory | F: 3x/week;  I: Moderate, Unknown;  T: <60 min;  T: Aerobic, Resistance;  D: 4 weeks |
| Merki-Künzli et al.; Assessing the value of prehabilitation in patients undergoing colorectal surgery according to the Enhanced Recovery After Surgery (ERAS) Pathway for the improvement of postoperative outcomes: protocol for a randomized controlled trial* | 2017 \| Switzerland \| RCT (protocol) \| SS: 112 \| RS: No | Supervised and home-based | Preoperatively |  | Surgical outcomes; CCI; 30 days postoperatively | Physical (A, B) | F: 3x/week;  I: High, Unknown;  T: >60 min;  T: Interval, Resistance;  D: 3 - 6 weeks |
| Groen et al.; Community-based prehabilitation in older patients and high-risk patients undergoing colorectal cancer surgery | 2024 \| The Netherlands \| RCT \| SS: 99 \| RS: Yes | Supervised and home-based | Preoperatively | Nutritional support; Psychosocial support; Smoking cessation; Medical optimization | Surgical outcomes; Clavien-Dindo; 90 days postoperatively | Physical (A, B, E) | F: >3x/week;  I: High, Moderate;  T: 60 min;  T: Interval, Resistance;  D: 3 weeks |
| Heil et al.; Improved Postoperative Outcomes after Prehabilitation for Colorectal Cancer Surgery in Older Patients: An Emulated Target Trial | 2023 \| The Netherlands \| Emulated target trial \| SS: 251 \| RS: Yes | Supervised and home-based | Preoperatively | Nutritional support | Surgical outcomes; Complication rate; Postoperatively |  | F: >3x/week;  I: Low (not specified), High;  T: 60 min;  T: Aerobic, Interval;  D: 3 weeks |
| Hogeweg-Raaijmakers et al.; A prehabilitation program for patients undergoing elective resection of a colorectal carcinoma: effects on the postoperative hospital stay and complication burden | 2022 \| The Netherlands \| Retrospective cohort \| SS: 282 \| RS: No | Supervised | Preoperatively | Nutritional support; Psychosocial support | Surgical outcomes; CCI and LOS; Postoperatively | Physical (A) | F: Unknown;  I: Unknown;  T: Unknown;  T: Unknown;  D: 3 - 6 weeks |
| Karlsson et al.; Feasibility of preoperative supervised home-based exercise in older adults undergoing colorectal cancer surgery - A randomized controlled design | 2019 \| Sweden \| RCT \| SS: 23 \| RS: No | Supervised | Preoperatively |  | Intervention Delivery; Feasibility assessed via recruitment rate, compliance to the intervention, and acceptability; Intervention period | Physical (A, B) | F: 3x/week;  I: High, High;  T: 60 min;  T: Aerobic, Resistance, IMT;  D: 2 weeks |
| Koh et al.; Structured presurgery prehabilitation for aged patients undergoing elective surgery significantly improves surgical outcomes and reduces cost: A nonrandomized sequential comparative prospective cohort study | 2022 \| Singapore \| Unrandomized controlled study \| SS: 58 \| RS: Yes | Home-based | Preoperatively | Nutritional support; Medical optimization | Surgical outcomes; LOS and morbidity and mortality; 30 days postoperatively | Physical (A, B, E) | F: Unknown;  I: Unknown;  T: Unknown;  T: Resistance;  D: 3 weeks |
| Li et al.; Impact of a trimodal prehabilitation program on Functional recovery after colorectal cancer surgery: a pilot study | 2013 \| Canada \| Unrandomized controlled study \| SS: 87 \| RS: No | Home-based | Preoperatively | Nutritional support; Psychosocial support | Physical or functional outcomes; 6MWT; 60 days postoperatively | Psychological; Physical (A, E); Metabolic; Inflammatory | F: 3x/week;  I: Moderate, Unknown;  T: <60 min;  T: Aerobic, Resistance;  D: 6 weeks |
| Lopez-Rodriguez-Arias et al.; Effect of home-based prehabilitation in an enhanced recovery after surgery program for patients undergoing colorectal cancer surgery during the COVID-19 pandemic | 2021 \| Spain \| RCT \| SS: 20 \| RS: No | Home-based | Preoperatively and postoperatively | Nutritional support | Physical or functional outcomes; Body composition (weight, lean mass, fat mass assessed via electrical impedance); Baseline, before surgery, 6 and 12 weeks postoperatively | Psychological; Physical (A, E) | F: >3x/week;  I: Unknown, Unknown;  T: <60 min;  T: Aerobic, Resistance, Relaxation, Breathing;  D: 4 weeks |
| Loughney et al.; Physical activity levels in locally advanced rectal cancer patients following neoadjuvant chemoradiotherapy and an exercise training programme before surgery: a pilot study | 2017 \| UK \| Unrandomized controlled study \| SS: 33 \| RS: No | Supervised | Preoperatively |  | Physical or functional outcomes; Change in physical activity assessed by the numbers of steps taken; Baseline, after NAC, before surgery | Physical (A) | F: 3x/week;  I: High;  T: <60 min;  T: Interval;  D: 6 weeks |
| Loughney et al.; The effects of neoadjuvant chemoradiotherapy and an in-hospital exercise training programme on physical fitness and quality of life in locally advanced rectal cancer patients (The EMPOWER Trial): study protocol for a randomised controlled trial* | 2016 \| UK \| RCT (protocol) \| SS: 46 \| RS: No | Supervised | Preoperatively |  | Physical or functional outcomes; Oxygen uptake at anaerobic threshold assessed via CPET; Baseline, after NAC, before surgery | Physical (A); Physiological | F: 3x/week;  I: High;  T: <60 min;  T: Interval;  D: 9 weeks |
| Macias-Valle et al.; Exercise effects on Functional capacity and quality of life in older patients with colorectal cancer: study protocol for the ECOOL randomized controlled trial | 2023 \| Spain \| RCT (protocol) \| SS: 250 \| RS: Yes | Home-based | Preoperatively and postoperatively |  | Physical or functional outcomes; Self-reported quality of life (EORTC-QLQ-C30); Baseline, discharge, 1, 3, 6 months postoperatively | Physical (A, B) | F: 1-2x/week;  I: Moderate, Moderate;  T: <60 min;  T: Aerobic, Resistance, Balance, IMT;  D: 2 weeks |
| van Rooijen et al.; Multimodal prehabilitation in colorectal cancer patients to improve Functional capacity and reduce postoperative complications: the first international randomized controlled trial for multimodal prehabilitation* | 2019 \| International \| RCT (protocol) \| SS: 714 \| RS: No | Supervised | Preoperatively | Nutritional support; Psychosocial support; Smoking cessation | Surgical outcomes; CCI; 30 days postoperatively | Psychological; Physical (A, B, E) | F: 3x/week;  I: High, Moderate;  T: 60 min;  T: Interval, Resistance;  D: 4 weeks |
| Moug et al.; Prehabilitation is feasible in patients with rectal cancer undergoing neoadjuvant chemoradiotherapy and may minimize physical deterioration: results from the REx trial | 2019 \| UK \| RCT \| SS: 48 \| RS: No | Home-based | Neoadjuvant treatment and preoperatively |  | Intervention Delivery; Feasibility/acceptability assessed by eligibility and recruitment rates, participant acceptability of randomization, data collection and physical intervention and rates of retention and adherence to the physical activity intervention; Intervention period | Physical (A, B, D, E) | F: >3x/week;  I: Low;  T: <60 min;  T: Aerobic;  D: 8 weeks |
| Northgraves et al.; Feasibility of a novel exercise prehabilitation programme in patients scheduled for elective colorectal surgery: a feasibility randomised controlled trial | 2020 \| UK \| RCT \| SS: 22 \| RS: No | Supervised | Preoperatively |  | Surgical outcomes; LOS; Postoperatively | Physical (B, D) | F: 3x/week;  I: Moderate, Unknown;  T: 60 min;  T: Aerobic, Resistance;  D: Unspecified |
| Onerup et al.; The effect of pre- and post-operative physical activity on recovery after colorectal cancer surgery (PHYSSURG-C): study protocol for a randomised controlled trial* | 2017 \| Sweden \| RCT (protocol) \| SS: 668 \| RS: No | Home-based | Preoperatively and postoperatively |  | Physical or functional outcomes; Patient-reported experience on physical recovery "To what extent do you feel fully physically recovered?"; 30 days postoperatively | Psychological; Physical (A, B); Physiological; Inflammatory | F: >3x/week;  I: Moderate (not specified);  T: <60 min;  T: Aerobic, IMT;  D: 2 weeks |
| Sabajo et al.; Prehabilitation in colorectal cancer surgery improves outcome and reduces hospital costs | 2024 \| The Netherlands \| Retrospective cohort \| SS: 586 \| RS: No | Supervised | Preoperatively | Nutritional support; Psychosocial support; Smoking cessation | Surgical outcomes; Clavien-Dindo, CCI; 30 days postoperatively |  | F: 3x/week;  I: NA, High, Moderate;  T: 60 min;  T: Interval, Resistance;  D: 4 weeks |
| Souwer et al.; Comprehensive multidiscipliry care program for elderly colorectal cancer patients: "From prehabilitation to independence" | 2018 \| The Netherlands \| Retrospective cohort \| SS: 224 \| RS: Yes | Supervised | Preoperatively | Nutritional support | Surgical outcomes; Mortality rate; 1 year postoperatively | Physical (A) | F: 1-2x/week;  I: Unknown, Unknown;  T: <60 min;  T: Aerobic, Resistance;  D: 4 - 6 weeks |
| Steffens et al.; Feasibility and acceptability of PrE-operative Physical Activity to improve patient outcomes After major cancer surgery: study protocol for a pilot randomised controlled trial (PEPA Trial)* | 2018 \| Australia \| RCT (protocol) \| SS: 20 \| RS: No | Supervised and home-based | Preoperatively |  | Intervention Delivery; Feasibility assessed via number of eligible patients recruited, retention and adherence rates; Intervention period |  | F: >3x/week;  I: Moderate, Moderate;  T: 60 min;  T: Aerobic, Resistance Stretching, Flexibility;  D: 2 - 6 weeks |
| Strijker et al.; Multimodal Prehabilitation in Patients Undergoing Complex Colorectal Surgery, Liver Resection, and Hyperthermic Intraperitoneal Chemotherapy (HIPEC): A Pilot Study on Feasibility and Potential Efficacy | 2023 \| The Netherlands \| Unrandomized controlled study \| SS: 63 \| RS: No | Supervised | Preoperatively | Nutritional support; Psychosocial support; Smoking cessation | Intervention Delivery; Feasibility assessed via accrual rate, completion rate, adherence, satisfaction and safety; Intervention period | Psychological; Physical (A, B, E); Metabolic | F: 3x/week;  I: High, Moderate;  T: 60 min;  T: Interval, Resistance;  D: Unspecified |
| Triguero-Canovas et al.; Home-based prehabilitation improves physical conditions measured by ergospirometry and 6MWT in colorectal cancer patients: a randomized controlled pilot study | 2023 \| Spain \| RCT \| SS: 44 \| RS: No | Home-based | Preoperatively and postoperatively | Nutritional support; Smoking cessation | Physical or functional outcomes; 6MWT and CPET; Baseline, before surgery, 6 weeks postoperatively | Psychological; Physical (A) | F: >3x/week;  I: Unknown, Unknown;  T: >60 min;  T: Aerobic, Resistance;  D: Unknown |
| van der Hulst et al.; Can physical prehabilitation prevent complications after colorectal cancer surgery in frail older patients? | 2021 \| The Netherlands \| Cohort study \| SS: 334 \| RS: Yes | Supervised | Preoperatively |  | Surgical outcomes; Rate of medical and surgical complications; 90 days postoperatively | Psychological; Inflammatory | F: 1-2x/week;  I: Unknown;  T: <60 min;  T: Unknown;  D: 4 weeks |
| van Rooijen et al.; Making Patients Fit for Surgery: Introducing a Four Pillar Multimodal Prehabilitation Program in Colorectal Cancer | 2019 \| The Netherlands \| Cohort study \| SS: 50 \| RS: No | Supervised | Preoperatively | Nutritional support; Psychosocial support; Smoking cessation | Intervention Delivery; Feasibility assessed via adherence, number of dropouts and safety; Intervention period | Physical (A) | F: 3x/week;  I: High, Moderate;  T: 60 min;  T: Interval, Resistance;  D: 4 weeks |
| Wang et al.; Effect of short-term prehabilitation of older patients with colorectal cancer: A propensity score-matched analysis | 2023 \| China \| Retrospective cohort \| SS: 190 \| RS: Yes | Supervised | Preoperatively | Nutritional support; Psychosocial support | Surgical outcomes; Clavien-Dindo, LOS, hospitalization costs, time to bowel function recovery, time to first ambulation, 30-day mortality, and 30-day hospital readmissions; 30 days postoperatively | Psychological; Physical (B, E); Metabolic | F: >3x/week;  I: High, Unknown;  T: >60 min;  T: Aerobic, Resistance, Breathing;  D: 1 week |
| West et al.; Effect of prehabilitation on objectively measured physical fitness after neoadjuvant treatment in preoperative rectal cancer patients: a blinded interventional pilot study | 2015 \| UK \| Cohort study \| SS: 35 \| RS: No | Supervised | Preoperatively |  | Physical or functional outcomes; Oxygen uptake and lactate threshold assessed via CPET; Baseline, before surgery, 6 weeks postoperatively | Physical (A) | F: 3x/week;  I: High;  T: <60 min;  T: Interval;  D: 6 weeks |
| Wong et al.; Evaluation of a physiatrist-directed prehabilitation intervention in frail patients with colorectal cancer: a randomised pilot study protocol | 2017 \| Canada \| RCT (protocol) \| SS: 50 \| RS: No | Supervised and home-based | Preoperatively | Psychosocial support | Intervention Delivery; Feasibility assessed via recruitment, refusal, retention, adherence, participant satisfaction and participant feedback; Intervention period | Physical (A) | F: 3x/week;  I: Moderate, Unknown;  T: Unknown;  T: Aerobic, Resistance, Flexibility;  D: 4 - 6 weeks |
| Fulop et al.; The effect of trimodal prehabilitation on the physical and psychological health of patients undergoing colorectal surgery: a randomised clinical trial. | 2021 \| Hungary \| RCT \| SS: 149 \| RS: No | Home-based | Preoperatively | Nutritional support; Psychosocial support | Physical or functional outcomes; 6MWT, respiratory reserve assessed via standard spirometry, self-reported quality of life (36-Item Short Form Survey) and Hospital and anxiety and depression assessed (Anxiety and Depression Scale); Baseline, presurgery, 4 and 8 weeks postoperatively | Psychological; Physical (A); Metabolic | F: >3x/week;  I: Low;  T: <60 min; T: Aerobic, Breathing;  D: 3 - 6 weeks |
| Carli et al.; Randomized clinical trial of prehabilitation in colorectal surgery. | 2010 \| Canada \| RCT \| SS: 112 \| RS: No | Home-based | Preoperatively |  | Physical or functional outcomes; 6MWT; Baseline, before surgery, 10 weeks postoperatively | Psychological; Physical (A, B) | F: >3x/week;  I: Low, Unknown;  T: <60 min;  T: Aerobic, Resistance;  D: 4 weeks |
| Morielli et al.; Exercise during and after neoadjuvant rectal cancer treatment (the EXERT trial): study protocol for a randomized controlled trial* | 2018 \| Canada \| RCT (protocol) \| SS: 60 \| RS: No | Home-based | Neoadjuvant treatment and preoperatively |  | Physical or functional outcomes; VO_2_ peak assessed using CPET; Baseline, after NAC, before surgery | Physical (A); Cardiovascular | F: 3x/week;  I: Moderate;  T: <60 min;  T: Aerobic;  D: 6 - 8 weeks |

**Study info:** RCT = Randomized controlled trial, SS = Sample size, RS = High-risk patient inclusion criteria;
**Main outcome:** CCI = Charlson Comorbidity Index, 6MWD= Six-minute walk test, LOS = Length of stay, AE = Adverse event, NAC = Neoadjuvant chemotherapy; CPET = Cardiopulmonary exercise test;
**Pathways:** A = Cardiovascular endurance, B = Muscular strength, C = Muscular endurance, D = Flexibility or mobility, E = Body composition;
**FITT and duration:** FITT = Frequency, intensity, time, type, IMT = Inspiratory muscle training, EMT = Expiratory muscle training;
Supervised was defined as >1 supervised session.
*Protocol and results paper were used. The protocol paper is presented.

1. **Lung Cancer**

| **Author and title** | **Study info** | **Delivery** | **Timing** | **Other components** | **Main outcome** | **Pathways** | **FITT and duration** |
| --- | --- | --- | --- | --- | --- | --- | --- |
| Barberan-Garcia et al.; Cost-effectiveness of a technology-supported multimodal prehabilitation program in moderate-to-high risk patients undergoing lung cancer resection: randomized controlled trial protocol | 2020 \| Spain \| RCT (protocol) \| SS: 158 \| RS: Yes | Supervised | Unknown | Nutritional support; Psychosocial support; Smoking cessation | Cost-effectiveness; Quadruple Aim approach: i) Patients’ and professionals’ experience outcomes (by means of standardized questionnaires, focus groups and structured interviews); ii) Population health-based outcomes (e.g. hospital length of stay, number and severity of postoperative complications, peak oxygen uptake and levels of systemic inflammation); and, iii) Healthcare costs.; 30 days postoperatively | Psychological; Physical (A, E); Inflammatory | F: 3x/week;  I: High (unspecified), Unknown;  T: <60 min;  T: Interval, Resistance;  D: Unknown |
| Benzo et al.; Preoperative Pulmonary rehabilitation before lung cancer resection: results from two randomized studies | 2011 \| UK \| RCT \| SS: 28 \| RS: Yes | Supervised | Preoperatively |  | Surgical outcomes; LOS, Pulmonary complications; Postoperatively | Physical (A, B) | F: >3x/week;  I: Unknown, Unknown;  T: Unknown;  T: Aerobic, Resistance, IMT, Breathing;  D: 1 - 4 weeks |
| Boujibar et al.; Impact of prehabilitation on morbidity and mortality after Pulmonary lobectomy by minimally invasive surgery: a cohort study | 2018 \| France \| Retrospective cohort \| SS: 34 \| RS: Yes | Supervised | Preoperatively | Smoking cessation; Therapeutic education | Surgical outcomes; Clavien-Dindo; 30 days postoperatively | Physical (A, B) | F: 3x/week or more;  I: Unknown, Moderate;  T: >60 min;  T: Aerobic, Resistance, IMT;  D: Unspecified |
| Bradley et al.; Pulmonary rehabilitation programme for patients undergoing curative lung cancer surgery | 2013 \| UK \| Cohort study \| SS: 363 \| RS: No | Supervised | Preoperatively | Nutritional support; Psychosocial support; Smoking cessation; Self-management education | Intervention Delivery; Feasibility, not specified; Intervention period | Physical (A, B) | F: 1-2x/week;  I: Moderate, NA, Moderate;  T: 60 min;  T: Aerobic, Resistance, IMT;  D: Unknown |
| Chen et al.; Effectiveness of precise and quantitative rapid Pulmonary rehabilitation nursing program for elderly patients with lung cancer during the perioperative period: A randomized controlled trial | 2023 \| China \| RCT \| SS: 218 \| RS: Yes | Supervised | Preoperatively and postoperatively |  | Surgical outcomes; Complication rate; Postoperatively | Psychological; Physical (A); Metabolic; Cardiovascular | F: Unknown;  I: NA;  T: Unknown;  T: Breathing;  D: 3 weeks |
| Ferreira et al.; Feasibility of a novel mixed-nutrient supplement in a multimodal prehabilitation intervention for lung cancer patients awaiting surgery: A randomized controlled pilot trial | 2021 \| Canada \| RCT \| SS: 34 \| RS: No | Supervised and home-based | Preoperatively | Nutritional support; Psychosocial support | Intervention Delivery; Feasibility based on recruitment and adherence rates; Intervention period | Psychological; Physical (A, E); Metabolic | F: 3x/week;  I: Moderate, Moderate;  T: 60 min;  T: Aerobic, Resistance, Relaxation;  D: 4 weeks |
| Ferreira et al.; Multimodal Prehabilitation for Lung Cancer Surgery: A Randomized Controlled Trial | 2021 \| Canada \| RCT \| SS: 95 \| RS: No | Home-based | Preoperatively | Nutritional support; Psychosocial support; Smoking cessation | Physical or functional outcomes; 6MWD; Baseline, before surgery, 4 and 8 weeks postoperatively | Psychological; Physical (A, E); Metabolic | F: 3x/week;  I: Unknown, Unknown;  T: <60 min;  T: Aerobic, Resistance;  D: 4 weeks |
| Fugazzaro et al.; PUREAIR protocol: randomized controlled trial of intensive Pulmonary rehabilitation versus standard care in patients undergoing surgical resection for lung cancer* | 2017 \| Italy \| RCT (protocol) \| SS: 140 \| RS: No | Supervised and home-based | Preoperatively and postoperatively |  | Physical or functional outcomes; 6MWD; Baseline, 6 months postoperatively | Physical (A, B) | F: >3x/week;  I: High, Moderate;  T: 60 min;  T: Aerobic, Resistance, IMT;  D: 2 - 3 weeks |
| Gao et al.; Cardiopulmonary exercise testing screening and pre-operative Pulmonary rehabilitation reduce postoperative complications and improve fast-track recovery after lung cancer surgery: A study for 342 cases | 2015 \| China \| Unrandomized controlled study \| SS: 142 \| RS: Yes | Supervised | Preoperatively |  | Surgical outcomes; Complication rate; Postoperatively | Physical (A, B); Physiological | F: >3x/week;  I: Moderate;  T: <60 min;  T: Aerobic, Breathing;  D: Unspecified |
| Huang et al.; Short-term high-intensity rehabilitation in radically treated lung cancer: a three-armed randomized controlled trial | 2017 \| China \| RCT \| SS: 90 \| RS: Yes | Supervised | Preoperatively |  | Surgical outcomes; Clavien-Dindo ≥2; 30 days postoperatively | Psychological; Physical (A, B) | F: >3x/week;  I: Unknown;  T: <60 min;  T: Aerobic, IMT, Breathing;  D: 1 week |
| Kong et al.; Perioperative Pulmonary rehabilitation training (PPRT) can reduce the cost of medical resources in patients undergoing thoracoscopic lung cancer resection: a retrospective study | 2021 \| China \| Retrospective cohort \| SS: 1427 \| RS: No | Unknown | Preoperatively |  | Cost-effectiveness; LOS, non-surgical expenses, and pulmonary complications; Postoperatively | Physical (B) | F: >3x/week;  I: Low, Unknown;  T: >60 min;  T: Aerobic, Resistance, IMT, Breathing, Flexibility;  D: Unknown |
| Lai et al.; Seven-day intensive preoperative rehabilitation for elderly patients with lung cancer: a randomized controlled trial | 2017 \| China \| RCT \| SS: 60 \| RS: No | Supervised | Preoperatively |  | Surgical outcomes; Clavien-Dindo ≥2; Postoperatively | Physical (A, B) | F: >3x/week;  I: Unknown;  T: <60 min;  T: Aerobic, EMT, Breathing;  D: 1 week |
| Lai et al.; Systematic short-term Pulmonary rehabilitation before lung cancer lobectomy: A randomized trial | 2017 \| China \| RCT \| SS: 101 \| RS: Yes | Supervised | Preoperatively |  | Surgical outcomes; Pulmonary complications; 30 days postoperatively | Physical (A, B) | F: >3x/week;  I: Unknown;  T: <60 min;  T: Aerobic, EMT, Breathing;  D: 1 week |
| Lai et al.; Impact of one-week preoperative physical training on clinical outcomes of surgical lung cancer patients with limited lung function: a randomized trial | 2019 \| China \| RCT \| SS: 68 \| RS: Yes | Supervised | Preoperatively |  | Physical or functional outcomes; 6MWT; Baseline, before surgery | Psychological; Physical (A); Cardiovascular | F: >3x/week;  I: Unknown;  T: <60 min;  T: Aerobic, Breathing;  D: 1 week |
| Laurent et al.; Preoperative respiratory muscle endurance training improves ventilatory capacity and prevents Pulmonary postoperative complications after lung surgery | 2020 \| France \| RCT \| SS: 26 \| RS: No | Supervised and home-based | Preoperatively |  | Physical or functional outcomes; Change in respiratory muscle endurance evaluated with the isocapnic hyperpnoea endurance test; Baseline, before surgery | Physical (A, B); Cardiovascular | F: >3x/week;  I: NA;  T: Unknown;  T: IMT;  D: 3 weeks |
| Laurent et al.; Effect of an intensive 3-week preoperative home rehabilitation programme in patients with chronic obstructive Pulmonary disease eligible for lung cancer surgery: a multicentre randomised controlled trial | 2017 \| France \| RCT \| SS: 90 \| RS: Yes | Supervised and home-based | Preoperatively |  | Surgical outcomes; Hospital discharge ability assessed via a 10-item list; 90 days postoperatively | Physical (A) | F: >3x/week;  I: High, Unknown;  T: <60 min;  T: Aerobic, Interval, Resistance;  D: 3 weeks |
| Lawson et al.; Effects of multimodal prehabilitation on muscle size, myosteatosis, and dietary intake of surgical patients with lung cancer - a randomized feasibility study | 2021 \| Canada \| RCT \| SS: 34 \| RS: No | Supervised and home-based | Preoperatively | Nutritional support; Psychosocial support; Smoking cessation | Intervention Delivery; Feasibility assessed via recruitment, eligibility, consent, attrition, acceptability of the outcome measures, and adherence rates; Intervention period | Psychological; Physical (E); Metabolic | F: >3x/week;  I: Moderate, Moderate;  T: 60 min;  T: Aerobic, Resistance;  D: 4 weeks |
| Licker et al.; Short-Term Preoperative High-Intensity Interval Training in Patients Awaiting Lung Cancer Surgery: A Randomized Controlled Trial | 2017 \| Switzerland \| RCT \| SS: 151 \| RS: No | Supervised | Preoperatively |  | Surgical outcomes; Composite end point of post-operative morbidity: 30-day mortality or complications with thoracic mortality and morbidity (TMM) ≥2; 30 days postoperatively | Physical (A); Physiological; Metabolic | F: 3x/week;  I: High;  T: <60 min;  T: Interval;  D: 2 - 3 weeks |
| Liu et al.; Two-Week Multimodal Prehabilitation Program Improves Perioperative Functional Capability in Patients Undergoing Thoracoscopic Lobectomy for Lung Cancer: A Randomized Controlled Trial | 2020 \| China \| RCT \| SS: 73 \| RS: No | Supervised | Preoperatively | Nutritional support; Psychosocial support | Physical or functional outcomes; 6MWT; Baseline, before surgery, 30 days postoperatively | Physical (A); Metabolic | F: 3x/week;  I: Moderate, Moderate;  T: 60 min;  T: Aerobic, Resistance, Breathing, Stretching;  D: 2 weeks |
| Lu et al.; Effects of Preoperative High-Intensity Interval Training Combined With Team Empowerment Education in Lung Cancer Patients With Surgery: A Quasi-experimental Trial | 2023 \| China \| Unrandomized controlled study \| SS: 83 \| RS: No | Supervised | Preoperatively | Team empowerment education | Physical or functional outcomes; Self-reported questionnaire British Medical Research Council Dyspnea Scale; Baseline, before surgery, discharge | Psychological; Physical (A) | F: >3x/week;  I: High;  T: <60 min;  T: Interval;  D: Unspecified |
| Machado et al.; Effect of Preoperative Home-Based Exercise Training on Quality of Life After Lung Cancer Surgery: A Multicenter Randomized Controlled Trial | 2024 \| Portugal \| RCT \| SS: 46 \| RS: No | Home-based | Preoperatively |  | Physical or functional outcomes; Self-reported quality of life (EORTC-QLQ-C30); Baseline, before surgery, 30 days postoperatively | Psychological; Physical (A, B) | F: 3x/week;  I: Low, Moderate;  T: 60 min;  T: Aerobic, Resistance;  D: 3 - 4 weeks |
| Minnella et al.; Prehabilitation in Thoracic Cancer Surgery: From Research to Standard of Care | 2021 \| Canada \| Implementation design \| SS: 81 \| RS: Yes | Supervised or home-based | Preoperatively | Nutritional support; Psychosocial support | Intervention Delivery; Feasibility and safety assessed via adherence and AE; Intervention period |  | F: 3x/week;  I: High;  T: Unknown;  T: Interval or aerobic;  D: 4 weeks |
| Molenaar et al.; Multimodal prehabilitation in patients with non-small cell lung cancer undergoing atomical resection: protocol of a non-randomised feasibility study | 2023 \| The Netherlands \| Unrandomized controlled study (protocol) \| SS: 40 \| RS: No | Supervised | Preoperatively | Nutritional support; Psychosocial support; Smoking cessation; Patient empowerment education | Intervention Delivery; Feasibility assessed via adherence; Intervention period | Physical (A, B) | F: 3x/week;  I: High, Moderate;  T: 60 min;  T: Interval, Resistance, IMT, Breathing;  D: 3 weeks |
| Patel et al.; Preconditioning program reduces the incidence of prolonged hospital stay after lung cancer surgery: Results from the Move For Surgery randomized clinical trial | 2023 \| Canada \| RCT \| SS: 95 \| RS: No | Home-based | Preoperatively | Nutritional support; Smoking cessation; Sleep optimization | Surgical outcomes; LOS; Postoperatively |  | F: >3x/week;  I: Moderate;  T: Unknown;  T: Aerobic, Breathing;  D: 3 - 4 weeks |
| Saito et al.; Impact of Pulmonary rehabilitation on postoperative complications in patients with lung cancer and chronic obstructive Pulmonary disease | 2017 \| Japan \| Retrospective cohort \| SS: 116 \| RS: Yes | Supervised and home-based | Preoperatively |  | Physical or functional outcomes; vital capacity, forced expiratory volume in 1 second assessed via CPET; Baseline, before surgery, 1 and 6 months postoperatively | Physical (A) | F: >3x/week;  I: Moderate, Unknown;  T: Unknown;  T: Aerobic, Breathing; D: 2 - 4 weeks |
| Saito et al.; The effect of home-based preoperative Pulmonary rehabilitation before lung resection: A retrospective cohort study | 2021 \| Japan \| Retrospective cohort \| SS: 144 \| RS: No | Supervised and home-based | Preoperatively |  | Surgical outcomes; Clavien-Dindo; Postoperatively | Physical (B); Physiological | F: >3x/week;  I: Unknown;  T: Unknown;  T: Breathing, Stretching;  D: 2 - 4 weeks |
| Sebio Garcia et al.; Preoperative exercise training prevents Functional decline after lung resection surgery: a randomized, single-blind controlled trial | 2017 \| Spain \| RCT \| SS: 22 \| RS: Yes | Supervised | Preoperatively |  | Physical or functional outcomes; Exercise capacity assessed via Constant-load Cycle Endurance Test performed at 80% of the peak workload; Baseline, 3 weeks postoperatively | Physical (A) | F: 3x/week or more;  I: High, Moderate;  T: 60 min;  T: Interval, Resistance, Breathing;  D: Unspecified |
| Sekine et al.; Perioperative rehabilitation and physiotherapy for lung cancer patients with chronic obstructive Pulmonary disease | 2005 \| Japan \| Retrospective cohort \| SS: 82 \| RS: Yes | Supervised and home-based | Preoperatively and postoperatively |  | Surgical outcomes; Pulmonary complications; Hospitalization period | Physical (B) | F: >3x/week;  I: Low;  T: 60 min;  T: Aerobic, Breathing;  D: 2 weeks |
| Sommer et al.; Perioperative rehabilitation in operation for lung cancer (PROLUCA) - rationale and design* | 2014 \| Denmark \| RCT (protocol) \| SS: 380 \| RS: No | Home-based | Preoperatively and postoperatively |  | Physical or functional outcomes; VO_2_ peak assessed via CPET; Baseline, before surgery, 4, 6 and 12 months postoperatively | Physical (A, B) | F: >3x/week;  I: Moderate;  T: <60 min;  T: Aerobic;  D: 2 weeks |
| Stefanelli et al.; High-intensity training and Cardiopulmonary exercise testing in patients with chronic obstructive Pulmonary disease and non-small-cell lung cancer undergoing lobectomy | 2013 \| Italy \| RCT \| SS: 40 \| RS: Yes | Supervised | Preoperatively |  | Physical or functional outcomes; VO_2_ peak assessed via CPET; Baseline, before surgery, 60 days postoperatively | Physical (A) | F: >3x/week;  I: High;  T: <60 min;  T: Aerobic, Breathing;  D: 3 weeks |
| Ulrich et al.; Precision-Exercise-Prescription in patients with lung cancer undergoing surgery: Rationale and design of the PEP study trial | 2018 \| USA \| RCT (protocol) \| SS: 200 \| RS: No | Home-based | Preoperatively |  | Physical or functional outcomes; 6MWT; Baseline, 60 days postoperatively | Physical (No) | F: >3x/week;  I: Moderate, Moderate;  T: <60 min;  T: Aerobic, Resistance, Mobility exercises;  D: Unknown |
| Zhou et al.; Short-term inpatient-based high-intensive Pulmonary rehabilitation for lung cancer patients: Is it feasible and effective? | 2017 \| China \| Retrospective cohort \| SS: 939 \| RS: Yes | Supervised | Preoperatively |  | Surgical outcomes; Clavien-Dindo (respiratory complications); 30 days postoperatively | Physical (A, B) | F: >3x/week;  I: Unknown;  T: <60 min;  T: Aerobic, IMT, Breathing;  D: 1 week |

**Study info:** RCT = Randomized controlled trial, SS = Sample size, RS = High-risk patient inclusion criteria;
**Main outcome:** CCI = Charlson Comorbidity Index, 6MWD= Six-minute walk test, LOS = Length of stay, AE = Adverse event, NAC = Neoadjuvant chemotherapy; CPET = Cardiopulmonary exercise test;
**Pathways:** A = Cardiovascular endurance, B = Muscular strength, C = Muscular endurance, D = Flexibility or mobility, E = Body composition;
**FITT and duration:** FITT = Frequency, intensity, time, type, IMT = Inspiratory muscle training, EMT = Expiratory muscle training;
Supervised was defined as >1 supervised session.
*Protocol and results paper were used. The protocol paper is presented.

1. **Upper GI Cancer**

| **Author and title** | **Study info** | **Delivery** | **Timing** | **Other components** | **Main outcome** | **Pathways** | **FITT and duration** |
| --- | --- | --- | --- | --- | --- | --- | --- |
| Allen et al.; A randomised controlled trial to assess whether prehabilitation improves fitness in patients undergoing neoadjuvant treatment prior to oesophagogastric cancer surgery: study protocol* | 2018 \| UK \| RCT (protocol) \| SS: 58 \| RS: No | Supervised and home-based | Neoadjuvant treatment and preoperatively | Nutritional support; Psychosocial support | Physical or functional outcomes; AT assessed via CPET; Baseline and before surgery | Psychological; Physical (A, B, E); Metabolic; Cardiovascular; Inflammatory | F: >3x/week;  I: Moderate, Moderate;  T: 60 min;  T: Aerobic, Resistance, Flexibility;  D: 6 weeks |
| Ausania et al.; Prehabilitation in patients undergoing pancreaticoduodenectomy: a randomized controlled trial | 2019 \| Spain \| RCT \| SS: 40 \| RS: No | Supervised and home-based | Preoperatively | Nutritional support | Surgical outcomes; Clavien-Dindo; Postoperatively | Physical (A) | F: >3x/week;  I: Unknown, Unknown;  T: 60 min;  T: Aerobic, Resistance, Breathing;  D: Unspecified |
| Bausys et al.; Personalized trimodal prehabilitation for gastrectomy* | 2020 \| Lithuania \| RCT (protocol) \| SS: 128 \| RS: No | Supervised and home-based | Neoadjuvant treatment and preoperatively | Nutritional support; Psychosocial support | Surgical outcomes; Clavien-Dindo; 90 days postoperatively | Psychological; Physical (A, E) | F: >3x/week;  I: Moderate, Unknown;  T: <60 min;  T: Aerobic, Resistance, IMT, Stretching;  D: 4 weeks |
| Chan et al.; Outcomes of an outpatient home-based prehabilitation program before pancreaticoduodenectomy: A retrospective cohort study | 2022 \| Singapore \| Cohort study \| SS: 71 \| RS: No | Home-based | Preoperatively |  | Surgical outcomes; Days, defined as presence of any postoperative morbidity such as clinically relevant postoperative pancreatic fistula, intra-abdominal collection, delayed gastric emptying, ileus, surgical site infection, pneumonia or pleural effusion; 30 days postoperatively | Physical (E) | F: >3x/week;  I: Moderate (not specified), Moderate (not specified);  T: <60 min;  T: Aerobic, Resistance, Breathing;  D: 2 - 4 weeks |
| Cho et al.; A randomized phase II trial of preoperative exercise to reduce operative risk in gastric cancer patients with metabolic syndrome: adjuvant exercise for general elective surgery (AEGES) study group* | 2008 \| Japan \| RCT (protocol) \| SS: 86 \| RS: Yes | Unknown | Preoperatively |  | Surgical outcomes; Proportion of postoperative complications; Postoperatively | Physical (E) | F: 3x/week or more;  I: Unknown, Unknown;  T: Unknown;  T: Aerobic, Resistance, Stretching;  D: 4 weeks |
| Christensen et al.; Safety and feasibility of preoperative exercise training during neoadjuvant treatment before surgery for adenocarcinoma of the gastro-oesophageal junction | 2019 \| Denmark \| Unrandomized controlled study \| SS: 50 \| RS: No | Supervised | Neoadjuvant treatment and preoperatively |  | Intervention Delivery; Feasibility/safety assessed via incidence of serious adverse events; Intervention period | Physical (A); Cardiovascular; Inflammatory | F: 1-2x/week;  I: High, High;  T: >60 min;  T: Interval, Resistance;  D: 4 - 6 weeks |
| Halliday et al.; The effects of prehabilitation on body composition in patients undergoing multimodal therapy for Oesophageal cancer | 2023 \| UK \| Retrospective cohort \| SS: 79 \| RS: No | Home-based | Neoadjuvant treatment and preoperatively | Nutritional support; Psychosocial support; Medical optimization | Physical or functional outcomes; Body composition (weight, body mass index, skeletal muscle, visceral adipose tissue, sub cutaneous adipose tissue, total adipose tissue, skeletal muscle index assessed via CT-scan L3); Baseline, before surgery | Physical (E); Inflammatory | F: >3x/week;  I: Moderate, Unknown;  T: >60 min;  T: Aerobic, Resistance;  D: Unknown |
| Halliday et al.; The Impact of Prehabilitation on Post-operative Outcomes in Oesophageal Cancer Surgery: a Propensity Score Matched Comparison | 2021 \| UK \| RCT \| SS: 111 \| RS: No | Home-based | Neoadjuvant treatment and preoperatively | Nutritional support; Psychosocial support; Medical optimization | Surgical outcomes; Complication rate; 60 days postoperatively | Psychological; Physical (A) | F: >3x/week;  I: Moderate, Unknown;  T: >60 min;  T: Aerobic, Resistance;  D: Unknown |
| Inoue et al.; Prevention of postoperative Pulmonary complications through intensive preoperative respiratory rehabilitation in patients with Oesophageal cancer | 2013 \| Japan \| RCT \| SS: 100 \| RS: No | Supervised and home-based | Preoperatively |  | Surgical outcomes; Pulmonary complications; 12 days postoperatively | Physical (B) | F: >3x/week;  I: Unknown, Unknown;  T: 60 min;  T: Aerobic, Resistance, IMT, Breathing;  D: 1 week |
| Janssen et al.; Effect of a multimodal prehabilitation program on postoperative recovery and morbidity in patients undergoing a totally minimally invasive esophagectomy | 2022 \| The Netherlands \| Retrospective cohort \| SS: 61 \| RS: No | Home-based | Neoadjuvant treatment and preoperatively | Nutritional support; Psychosocial support | Physical or functional outcomes; Functional recovery was defined as: adequate pain control with oral analgesics (Numeric Rating Scale/Visual Analog Scale 50% of calculated calories, no intravenous fluids, mobility restored to an independent level (e.g. walking to toilet) and no signs of an active infection (no fever and declining serum levels of C-reactive protein and leucocyte count); 30 days postoperatively | Physical (A, B) | F: >3x/week;  I: Low;  T: 60 min;  T: Aerobic, Resistance, IMT;  D: Unknown |
| Jianjun et al.; Application of Exercised-based Pre-rehabilitation in Perioperative Period of Patients with Gastric Cancer | 2019 \| China \| RCT \| SS: 120 \| RS: No | Unknown | Preoperatively | Nutritional support | Surgical outcomes; LOS; Postoperatively | Psychological; Physical (E); Metabolic; Inflammatory | F: >3x/week;  I: Low, Unknown;  T: Unknown;  T: Aerobic, Resistance, Breathing;  D: 1 week |
| Mazzola et al.; Frailty in major oncologic surgery of upper Gastrointestinal tract: How to improve postoperative outcomes | 2017 \| Italy \| Unrandomized controlled study \| SS: 76 \| RS: Yes | Home-based | Preoperatively | Nutritional support; Smoking cessation | Surgical outcomes; 90-day mortality rates; 90 days postoperatively | Physical (A, B) | F: 3x/week;  I: Moderate;  T: <60 min;  T: Aerobic, Breathing;  D: Unknown |
| Minnella et al.; Effect of Exercise and Nutrition Prehabilitation on Functional Capacity in Esophagogastric Cancer Surgery: A Randomized Clinical Trial | 2018 \| Canada \| RCT \| SS: 51 \| RS: No | Home-based | Preoperatively | Nutritional support | Physical or functional outcomes; 6MWT; Baseline, before surgery, 4 and 8 weeks postoperatively | Physical (A) | F: >3x/week;  I: Moderate, Moderate;  T: 60 min;  T: Aerobic, Resistance;  D: Unspecified |
| Nakajima et al.; Clinical Benefit of Preoperative Exercise and nutritional Therapy for Patients Undergoing Hepato-Pancreato-Biliary Surgeries for Malignancy | 2019 \| Japan \| Unrandomized controlled study \| SS: 152 \| RS: No | Home-based | Preoperatively | Nutritional support | Physical or functional outcomes; Nutritional status (body weight, body mass index, serum albumin, prognostic nutritional index); Baseline, before surgery | Physical (A, E) | F: >3x/week;  I: Low, Unknown;  T: 60 min;  T: Aerobic, Resistance;  D: Unspecified |
| Ngo-Huang et al.; Effects of a Pragmatic Home-based Exercise Program Concurrent With Neoadjuvant Therapy on Physical Function of Patients With Pancreatic Cancer: The PancFit Randomized Clinical Trial | 2023 \| USA \| RCT \| SS: 125 \| RS: No | Home-based | Preoperatively |  | Physical or functional outcomes; 6MWT; Baseline, before surgery | Physical (A, E) | F: >3x/week;  I: Moderate, Unknown;  T: 60 min;  T: Aerobic, Resistance, Stretching; D: 6 weeks |
| Parker et al.; The Role of Home-Based Exercise in Maintaining Skeletal Muscle During Preoperative Pancreatic Cancer Treatment | 2021 \| USA \| Unrandomized controlled study \| SS: 97 \| RS: No | Home-based | Preoperatively |  | Physical or functional outcomes; Skeletal muscle mass assessed via CT-scan; Baseline, before surgery | Physical (A, E) | F: 3x/week;  I: Low, Moderate;  T: 60 min;  T: Aerobic, Resistance;  D: 6 weeks |
| Pfirrmann et al.; Internet-based perioperative exercise program in patients with Barrett's carcinoma scheduled for esophagectomy [iPEP - study] a prospective randomized-controlled trial | 2017 \| Germany \| RCT \| SS: 80 \| RS: No | Home-based | Preoperatively and postoperatively |  | Physical or functional outcomes; Peak oxygen uptake assessed via CPET; Baseline, before surgery | Physical (B) | F: >3x/week;  I: Unknown, Moderate;  T: 60 min;  T: Aerobic, Resistance, Breathing;  D: Unknown |
| Steffens et al.; PRehabIlitatiOn with pReoperatIve exercise and educaTion for patients undergoing major abdominal cancer surgerY: protocol for a multicentre randomised controlled TRIAL (PRIORITY TRIAL) | 2022 \| Australia \| RCT (protocol) \| SS: 172 \| RS: No | Supervised | Preoperatively |  | Surgical outcomes; Complication rate; Hospitalization period | Physical (A, B) | F: >3x/week;  I: Low, High, Moderate;  T: <60 min;  T: Aerobic, Interval, Resistance, Breathing;  D: 4 - 8 weeks |
| Sun et al.; Multimodal prehabilitation to improve the clinical outcomes of frail elderly patients with gastric cancer: a study protocol for a multicentre randomised controlled trial (GISSG+2201) | 2023 \| China \| RCT (protocol) \| SS: 368 \| RS: Yes | Supervised | Preoperatively | Nutritional support; Psychosocial support; Smoking cessation | Surgical outcomes; Clavien-Dindo; 30 days postoperatively | Psychological; Physical (A, B, E); Metabolic; Inflammatory | F: 3x/week or more;  I: Moderate, Unknown;  T: 60 min;  T: Aerobic, Resistance, IMT;  D: 2 weeks |
| Tully et al.; The effect of a pre- and post-operative exercise programme versus standard care on physical fitness of patients with oesophageal and gastric cancer undergoing neoadjuvant treatment prior to surgery (The PERIOP-OG Trial): Study protocol for a randomised controlled trial | 2020 \| Ireland \| RCT (protocol) \| SS: 62 \| RS: No | Supervised or home-based | Preoperatively and postoperatively |  | Physical or functional outcomes; 6MWT; Baseline, before surgery | Physical (A, B) | F: 3x/week;  I: Moderate, High, Unknown;  T: <60 min;  T: Aerobic or Interval, Resistance;  D: 3 - 8 weeks |
| Valkenet et al.; Preoperative inspiratory muscle training to prevent postoperative pulmonary complications in patients undergoing oesophageal resection (PREPARE study): study protocol for a randomized controlled trial* | 2014 \| The Netherlands \| RCT (protocol) \| SS: 248 \| RS: No | Home-based | Preoperatively |  | Surgical outcomes; Postoperative pneumonia assessed via the Utrecht Pneumonia Scoring System; Postoperatively | Physical (A, B); Physiological | F: >3x/week;  I: NA;  T: Unknown;  T: IMT;  D: 2 - 6 weeks |
| Wada et al.; Preoperative nutrition and exercise intervention in frailty patients with gastric cancer undergoing gastrectomy | 2022 \| Japan \| Cohort study \| SS: 58 \| RS: Yes | Home-based | Preoperatively | Nutritional support | Surgical outcomes; Clavien-Dindo; Postoperatively | Inflammatory | F: >3x/week;  I: Low, Unknown;  T: <60 min;  T: Aerobic, Resistance;  D: Unspecified |
| Yamana et al.; Randomized Controlled Study to Evaluate the Efficacy of a Preoperative Respiratory Rehabilitation Program to Prevent Postoperative Pulmonary Complications after Esophagectomy | 2015 \| Japan \| RCT \| SS: 60 \| RS: No | Supervised | Preoperatively |  | Surgical outcomes; Clavien-Dindo (respiratory complications) and Utrecht Pneumonia Scoring System; Postoperatively | Physical (A, B); Coagulation | F: >3x/week;  I: Unknown, Unknown;  T: NA;  T: Aerobic, Resistance, Breathing;  D: Unspecified |
| Chmelo et al.; A feasibility study to investigate the utility of a home-based exercise intervention during and after neo-adjuvant chemotherapy for oesophago-gastric cancer-the ChemoFit study protocol | 2020 \| UK \| Unrandomized controlled study (protocol) \| SS: 40 \| RS: No | Home-based | Neoadjuvant treatment and preoperatively |  | Intervention Delivery; Feasibility assessed via recruitment, completion, compliance; Intervention period | Physical (A, B, E); Inflammatory | F: >3x/week;  I: Low, Unknown;  T: <60 min;  T: Aerobic, Resistance;  D: Unknown |
| Dettling et al.; Feasibility and effectiveness of pre-operative inspiratory muscle training in patients undergoing oesophagectomy: a pilot study | 2013 \| The Netherlands \| Cohort study \| SS: 78 \| RS: No | Supervised and home-based | Preoperatively |  | Intervention Delivery; Feasibility assessed via AE, patient satisfaction; Intervention period | Physical (A, B) | F: >3x/week;  I: NA;  T: Unknown;  T: IMT, Breathing;  D: 2 weeks |
| Kitahata et al.; Intensive perioperative rehabilitation improves surgical outcomes after pancreaticoduodenectomy. | 2018 \| Japan \| Cohort study \| SS: 576 \| RS: No | Supervised | Preoperatively and postoperatively |  | Surgical outcomes; Severe complications Clavien-Dindo >3, Pancreatic fistula Grade B and C, Pulmonary complications, Complications, Mortality, LOS; 30 days postoperatively | Physical (A, B); Cardiovascular; Inflammatory | F: >3x/week;  I: High, High;  T: >60 min;  T:Aerobic, Resistance, Breathing;  D: 1 week |

**Study info:** RCT = Randomized controlled trial, SS = Sample size, RS = High-risk patient inclusion criteria;
**Main outcome:** CCI = Charlson Comorbidity Index, 6MWD= Six-minute walk test, LOS = Length of stay, AE = Adverse event, NAC = Neoadjuvant chemotherapy; CPET = Cardiopulmonary exercise test;
**Pathways:** A = Cardiovascular endurance, B = Muscular strength, C = Muscular endurance, D = Flexibility or mobility, E = Body composition;
**FITT and duration:** FITT = Frequency, intensity, time, type, IMT = Inspiratory muscle training, EMT = Expiratory muscle training;
Supervised was defined as >1 supervised session.
*Protocol and results paper were used. The protocol paper is presented.

1. **Urological Cancer**

| **Author and title** | **Study info** | **Delivery** | **Timing** | **Other components** | **Main outcome** | **Pathways** | **FITT and duration** |
| --- | --- | --- | --- | --- | --- | --- | --- |
| Akdemir et al.; EffectiveNess of a multimodal preHAbilitation program in patieNts with bladder canCEr undergoing radical cystectomy: protocol of the ENHANCE multicentre randomised controlled trial | 2023 \| The Netherlands \| RCT (protocol) \| SS: 154 \| RS: No | Supervised | Neoadjuvant treatment and preoperatively | Nutritional support; Psychosocial support; Smoking cessation | Surgical outcomes; Clavien-Dindo ≥2; 90 days postoperatively | Psychological; Physical (A, B, E); Metabolic; Cardiovascular; Inflammatory | F: 3x/week;  I: High, Moderate;  T: 60 min;  T: Interval, Resistance, Relaxation;  D: 3 - 6 weeks |
| Aldhaam et al.; Impact of Perioperative Multidiscipliry Rehabilitation Pathway on Early Outcomes after Robot-assisted Radical Cystectomy: A Matched Analysis | 2021 \| USA \| Retrospective cohort \| SS: 192 \| RS: No | Home-based | Preoperatively | Nutritional support; Psychosocial support; Educational materials | Surgical outcomes; Clavien-Dindo; 30 days postoperatively | Physical (A) | F: 3x/week;  I: Unknown, Unknown;  T: 60 min;  T: Aerobic, Resistance;  D: 4 weeks |
| Banerjee et al.; Vigorous intensity aerobic interval exercise in bladder cancer patients prior to radical cystectomy: a feasibility randomised controlled trial | 2018 \| UK \| RCT \| SS: 60 \| RS: No | Supervised | Preoperatively |  | Intervention Delivery; Assessed by recruitment and attrition, willingness to be randomized, acceptability of the outcome measures, adherence to the intervention, safety and suitability of the exercise dose and adverse events; Intervention period | Physical (A); Metabolic | F: 1-2x/week;  I: High;  T: 60 min;  T: Interval;  D: 3 - 6 weeks |
| Blackwell et al.; High-intensity interval training produces a significant improvement in fitness in less than 31 days before surgery for urological cancer: a randomised control trial | 2020 \| UK \| RCT \| SS: 40 \| RS: No | Supervised | Preoperatively |  | Physical or functional outcomes; VO_2_ at improvement of 1,5-2 ml/kg/min assessed via CPET; Baseline and before surgery | Physical (A); Cardiovascular | F: >3x/week;  I: High;  T: <60 min;  T: Interval;  D: 4 weeks |
| Jensen et al.; Efficacy of a multiprofessional rehabilitation programme in radical cystectomy pathways: a prospective randomized controlled trial | 2015 \| Denmark \| RCT \| SS: 107 \| RS: No | Home-based | Preoperatively and postoperatively |  | Surgical outcomes; LOS; 30 days postoperatively | Physical (A) | F: >3x/week;  I: Unknown, Unknown;  T: Unknown;  T: Aerobic, Resistance;  D: 2 weeks |
| Minnella et al.; Multimodal Prehabilitation to Enhance Functional Capacity Following Radical Cystectomy: A Randomized Controlled Trial | 2021 \| Canada \| RCT \| SS: 70 \| RS: No | Home-based | Preoperatively | Nutritional support; Psychosocial support | Physical or functional outcomes; 6MWT; Baseline, before surgery, 4 and 8 weeks postoperatively | Psychological; Physical (A, B, E) | F: 3x/week;  I: Moderate, Moderate;  T: 60 min;  T: Aerobic, Resistance;  D: Unspecified |
| Tonnesen et al.; STRONG for Surgery & Strong for Life - against all odds: intensive prehabilitation including smoking, nutrition, alcohol and physical activity for risk reduction in cancer surgery - a protocol for an RCT with nested interview study (STRONG-Cancer) | 2022 \| Denmark \| RCT (protocol) \| SS: 42 \| RS: Yes | Home-based | Preoperatively | Nutritional support; Smoking cessation | Physical or functional outcomes; Number of patients with surgical risk reduction of at least 1 step for 1 or more risky lifestyles on the American Society of Anesthesiologists score; Baseline, before surgery | Physiological | F: >3x/week;  I: Unknown;  T: <60 min;  T: Unknown;  D: 6 weeks |

**Study info:** RCT = Randomized controlled trial, SS = Sample size, RS = High-risk patient inclusion criteria;
**Main outcome:** CCI = Charlson Comorbidity Index, 6MWD= Six-minute walk test, LOS = Length of stay, AE = Adverse event, NAC = Neoadjuvant chemotherapy; CPET = Cardiopulmonary exercise test;
**Pathways:** A = Cardiovascular endurance, B = Muscular strength, C = Muscular endurance, D = Flexibility or mobility, E = Body composition;
**FITT and duration:** FITT = Frequency, intensity, time, type, IMT = Inspiratory muscle training, EMT = Expiratory muscle training;
Supervised was defined as >1 supervised session.
*Protocol and results paper were used. The protocol paper is presented.

1. **Prostate Cancer**

| **Author and title** | **Study info** | **Delivery** | **Timing** | **Other components** | **Main outcome** | **Pathways** | **FITT and duration** |
| --- | --- | --- | --- | --- | --- | --- | --- |
| Bales et al.; Effect of preoperative biofeedback/pelvic floor training on continence in men undergoing radical prostatectomy | 2000 \| USA \| RCT \| SS: 100 \| RS: No | Home-based | Preoperatively |  | Physical or functional outcomes; Patients wearing one pad or less per day versus two or more pads per day; 30, 60, 90, 120, 180 days postoperatively | Physical (C) | F: >3x/week;  I: NA;  T: Unknown;  T: Pelvic muscle exercises with biofeedback;  D: 2 - 4 weeks |
| Kaushik et al.; Effects of yoga in men with prostate cancer on quality of life and immune response: a pilot randomized controlled trial | 2022 \| USA \| RCT \| SS: 26 \| RS: No | Supervised | Preoperatively and postoperatively |  | Physical or functional outcomes; Self-reported quality of life (Functional Assessment of Cancer Therapy-Prostate); Baseline, before surgery, 6 weeks postoperatively | Psychological; Physical (A, D); Inflammatory | F: 1-2x/week;  I: NA;  T: 60 min;  T: Hatha yoga (including breathing and pelvic floor engagement);  D: 6 weeks |
| Khorrami et al.; Single session pre-operative pelvic floor muscle training with biofeedback on Urinary incontinence and quality of life after radical prostatectomy: A randomized controlled trial | 2023 \| Canada \| RCT \| SS: 80 \| RS: No | Home-based | Preoperatively |  | Physical or functional outcomes; Self-reported urinary incontinence (ICIQ-UI); 1, 3 and 6 months after urinary catheter removal | Physical (B) | F: >3x/week; I: NA;  T: <60 min;  T: Pelvic muscle exercises with biofeedback;  D: 4 weeks |
| Patel et al.; Preoperative pelvic floor physiotherapy improves continence after radical retropubic prostatectomy | 2013 \| Australia \| Retrospective cohort \| SS: 284 \| RS: No | Supervised and home-based | Preoperatively and postoperatively |  | Physical or functional outcomes; Urinary incontinence assessed via 24-h pad weight; 45 and 90 days postoperatively | Physical (C) | F: >3x/week;  I: NA;  T: Unknown;  T: Pelvic muscle training;  D: 4 weeks |
| Santa Mina et al.; Prehabilitation for men undergoing radical prostatectomy: a multi-centre, pilot randomized controlled trial* | 2014 \| Canada \| RCT (protocol) \| SS: 100 \| RS: No | Home-based | Preoperatively |  | Intervention Delivery; Feasibility assessed via recruitment rate, adherence/ contamination, attrition, and safety; Intervention period | Psychological; Physical (A, C) | F: 3x/week or more;  I: Moderate, Unknown;  T: 60 min;  T: Aerobic, Resistance, Pelvic muscle training (pelvic muscle training also in control group);  D: 4 - 8 weeks |
| Schulz et al.; Taking Advantage of the Teachable Moment at Initial Diagnosis of Prostate Cancer-Results of a Pilot Randomized Controlled Trial of Supervised Exercise Training | 2022 \| Canada \| RCT \| SS: 19 \| RS: No | Supervised | Preoperatively |  | Patient Reported/Related Outcomes; Self-reported physical activity (Godin Leisure Time Physical Activity); Baseline, 6 months postoperatively |  | F: 1-2x/week;  I: Moderate, Moderate;  T: 60 min;  T: Aerobic, Resistance;  D: 8 - 12 weeks |
| Singh et al.; Prehabilitative versus rehabilitative exercise in prostate cancer patients undergoing prostatectomy | 2023 \| Australia \| RCT \| SS: 38 \| RS: No | Supervised | Preoperatively |  | Physical or functional outcomes; Muscle strength for chest press and leg press using 1-Repitition Maximum testing; Baseline, before surgery, 6 weeks and 12 weeks postoperatively | Physical (B) | F: 3x/week;  I: High, Moderate;  T: >60 min;  T: Aerobic, Resistance;  D: 6 weeks |

**Study info:** RCT = Randomized controlled trial, SS = Sample size, RS = High-risk patient inclusion criteria;
**Main outcome:** CCI = Charlson Comorbidity Index, 6MWD= Six-minute walk test, LOS = Length of stay, AE = Adverse event, NAC = Neoadjuvant chemotherapy; CPET = Cardiopulmonary exercise test;
**Pathways:** A = Cardiovascular endurance, B = Muscular strength, C = Muscular endurance, D = Flexibility or mobility, E = Body composition;
**FITT and duration:** FITT = Frequency, intensity, time, type, IMT = Inspiratory muscle training, EMT = Expiratory muscle training;
Supervised was defined as >1 supervised session.
*Protocol and results paper were used. The protocol paper is presented.

1. **Gynecological Cancer**

| **Author and title** | **Study info** | **Delivery** | **Timing** | **Other components** | **Main outcome** | **Pathways** | **FITT and duration** |
| --- | --- | --- | --- | --- | --- | --- | --- |
| Diaz-Feijoo et al.; A multimodal prehabilitation program for the reduction of post-operative complications after surgery in advanced ovarian cancer under an ERAS pathway: a randomized multicenter trial (SOPHIE) | 2022 \| Spain \| RCT (protocol) \| SS: 146 \| RS: No | Supervised | Preoperatively | Nutritional support; Psychosocial support | Surgical outcomes; Proportion of postoperative complications; 30 days postoperatively | Psychological; Physical (No); Metabolic | F: 3x/week;  I: High, Unknown;  T: Unknown;  T: Interval, Resistance, IMT;  D: 2 - 4 weeks |
| Diaz-Feijoo et al.; Feasibility of a Multimodal Prehabilitation Programme in Patients Undergoing Cytoreductive Surgery for Advanced Ovarian Cancer: A Pilot Study | 2022 \| Spain \| Unrandomized controlled study \| SS: 34 \| RS: No | Supervised | Preoperatively | Nutritional support; Psychosocial support | Intervention Delivery; Feasibility assessed via adherence; Intervention period | Psychological; Physical (E); Metabolic | F: 3x/week;  I: High, Unknown;  T: Unknown;  T: Interval, Resistance, IMT;  D: 2 - 4 weeks |
| Lopes et al.; PROPER-PRehabilitatiOn Plus Enhanced Recovery after surgery versus enhanced recovery after surgery in gynecologic oncology: a randomized clinical trial | 2022 \| Brazil \| RCT (protocol) \| SS: 194 \| RS: No | Supervised | Preoperatively | Nutritional support; Psychosocial support | Surgical outcomes; LOS; Postoperatively | Physical (A) | F: 3x/week;  I: Unknown, Unknown;  T: <60 min;  T: Aerobic, Resistance, IMT, Stretching;  D: 2 - 3 weeks |
| Miralpeix et al.; Impact of prehabilitation during neoadjuvant chemotherapy and interval cytoreductive surgery on ovarian cancer patients: a pilot study | 2022 \| Spain \| Retrospective cohort \| SS: 29 \| RS: No | Supervised or home-based | Neoadjuvant treatment and preoperatively | Nutritional support; Psychosocial support | Physical or functional outcomes; Nutritional parameters (total protein, albumin, prealbumin hemoglobin, preoperative HbA1c, intraoperative glucose, postoperative insulin requirement); Baseline, before surgery, 4 weeks postoperatively | Psychological; Physical (B, E); Metabolic | F: >3x/week;  I: Low, Unknown;  T: Unknown;  T: Aerobic, Resistance, Flexibility, IMT;  D: Unknown |
| Miralpeix et al.; Prehabilitation in an ERAS program for endometrial cancer patients: impact on post-operative recovery | 2023 \| Spain \| Retrospective cohort \| SS: 128 \| RS: No | Supervised or home-based | Preoperatively | Nutritional support; Psychosocial support; Medical optimization | Surgical outcomes; LOS; 30 days postoperatively | Metabolic | F: >3x/week;  I: Low, Unknown;  T: Unknown; T: Aerobic, Resistance, Flexibility, IMT;  D: 2 weeks |
| Xia et al.; Effects of preoperative walking on bowel function recovery for patients undergoing gynecological malignancy laparoscopy | 2022 \| China \| RCT \| SS: 156 \| RS: No | Home-based | Preoperatively |  | Surgical outcomes; Gastrointestinal function defined as the first postoperative exhaust and defecations time; Postoperatively | Physiological; Cardiovascular | F: >3x/week;  I: Low;  T: <60 min;  T: Aerobic; D: 1 week |
| Lambaudie et al.; TRAINING-Ovary 01 (connecTed pRehabiliAtIoN pelvIc caNcer surGery): multicenter randomized study comparing neoadjuvant chemotherapy for patients managed for ovarian cancer with or without a connected pre-habilitation program | 2021 \| France \| RCT \| SS: 136 \| RS: No | Home-based | Neoadjuvant treatment and preoperatively | Nutritional support; Psychosocial support | Physical or functional outcomes; VO_2_ peak assessed via CPET; Baseline, before surgery | Psychological; Physical (A) | F: >3x/week;  I: Unknown;  T: Unknown;  T: Unknown;  D: Unknown |

**Study info:** RCT = Randomized controlled trial, SS = Sample size, RS = High-risk patient inclusion criteria;
**Main outcome:** CCI = Charlson Comorbidity Index, 6MWD= Six-minute walk test, LOS = Length of stay, AE = Adverse event, NAC = Neoadjuvant chemotherapy; CPET = Cardiopulmonary exercise test;
**Pathways:** A = Cardiovascular endurance, B = Muscular strength, C = Muscular endurance, D = Flexibility or mobility, E = Body composition;
**FITT and duration:** FITT = Frequency, intensity, time, type, IMT = Inspiratory muscle training, EMT = Expiratory muscle training;
Supervised was defined as >1 supervised session.

1. **Breast Cancer**

| **Author and title** | **Study info** | **Delivery** | **Timing** | **Other components** | **Main outcome** | **Pathways** | **FITT and duration** |
| --- | --- | --- | --- | --- | --- | --- | --- |
| Baima et al.; Teaching of Independent Exercises for Prehabilitation in Breast Cancer | 2017 \| USA \| Unrandomized controlled study \| SS: 60 \| RS: No | Home-based | Preoperatively |  | Physical or functional outcomes; 11 point pain scale (0-10), range of motion (0 to 180 degrees), chart documentation of seroma formation; 90 days postoperatively | Physical (B, D) | F: >3x/week;  I: NA;  T: Unknown;  T: Shoulder exercise;  D: 1 - 4 weeks |
| Casanovas-Alvarez et al.; Prehabilitation in Patients With Breast Cancer Receiving Neoadjuvant Therapy to Minimize Musculoskeletal Postoperative Complications and Enhance Recovery (PREOPtimize): A Protocol for a Randomized Controlled Trial | 2023 \| Spain \| RCT (protocol) \| SS: 64 \| RS: No | Supervised | Preoperatively |  | Physical or functional outcomes; Functionality of the ipsilateral arm assessed via QuickDash questionnaire; Baseline, before surgery, 4 and 12 weeks postoperatively | Psychological; Physical (B, D) | F: 1-2x/week;  I: Moderate, Moderate;  T: >60 min;  T: Aerobic, Resistance, Breathing;  D: 6 - 9 weeks |
| Heiman et al.; Recovery after breast cancer surgery following recommended pre and postoperative physical activity: (PhysSURG-B) randomized clinical trial | 2021 \| Sweden \| RCT \| SS: 400 \| RS: No | Home-based | Preoperatively and postoperatively |  | Physical or functional outcomes; Physical recovery assessed via self-administered questionnaire: "To what extent do you feel physically recovered after surgery?"; 30 days postoperatively |  | F: >3x/week;  I: Moderate (not specified);  T: <60 min;  T: Aerobic;  D: 2 weeks |
| Wu et al.; The Feasibility of Prehabilitation as Part of the Breast Cancer Treatment Pathway | 2021 \| UK \| Cohort study \| SS: 44 \| RS: No | Supervised | Preoperatively | Nutritional support; Psychosocial support; Smoking cessation | Intervention Delivery; The feasibility assessed via number of patients who wanted to access the service, acceptance assessed via number of patients opting to participate, and patient satisfaction via service evaluation forms; Intervention period | Psychological; Physical (B); Metabolic | F: 1-2x/week;  I: Unknown;  T: <60 min;  T: Resistance;  D: 1 - 7 weeks |

**Study info:** RCT = Randomized controlled trial, SS = Sample size, RS = High-risk patient inclusion criteria;
**Main outcome:** CCI = Charlson Comorbidity Index, 6MWD= Six-minute walk test, LOS = Length of stay, AE = Adverse event, NAC = Neoadjuvant chemotherapy; CPET = Cardiopulmonary exercise test;
**Pathways:** A = Cardiovascular endurance, B = Muscular strength, C = Muscular endurance, D = Flexibility or mobility, E = Body composition;
**FITT and duration:** FITT = Frequency, intensity, time, type, IMT = Inspiratory muscle training, EMT = Expiratory muscle training;
Supervised was defined as >1 supervised session.

1. **Other Cancer / Mixed-Cancer**

| **Author and title** | **Study info** | **Delivery** | **Timing** | **Other components** | **Main outcome** | **Pathways** | **FITT and duration** |
| --- | --- | --- | --- | --- | --- | --- | --- |
| ***Colorectal or GI cancer or Upper abdominal*** | | | | | | | |
| Janssen et al.; A multicomponent prehabilitation pathway to reduce the incidence of delirium in elderly patients in need of major abdominal surgery: study protocol for a before-and-after study* | 2019 \| The Netherlands \| Unrandomized controlled study (protocol) \| SS: 550 \| RS: Yes | Home-based | Preoperatively | Nutritional support; Medical optimization | Physical or functional outcomes; Incidence of delirium screened with Delirium Observational Screening Score, score of ≥3. Confirmed with DSM-V criteria and confusion assessment method.; Hospitalization period | Psychological; Physical (A, B, E); Metabolic | F: >3x/week;  I: Low, Unknown;  T: 60 min;  T: Aerobic, Resistance, IMT;  D: 4 - 5 weeks |
| ***Upper GI or Colorectal cancer*** | | | | | | | |
| Li et al.; Effect of trimodal prerehabilitation on the rehabilitation of patients with Gastrointestinal tumors in the perioperative period | 2022 \| China \| Retrospective cohort \| SS: 878 \| RS: No | Supervised | Preoperatively | Nutritional support; Psychosocial support | Physical or functional outcomes; Nutritional status assessed via prealbumin and albumin levels, self-reported sleep quality (Pittsburgh Sleep Quality Index) and psychological status (depression scale and anxiety scale), 6MWD and grip strength ; Baseline, before surgery, at discharge | Psychological; Physical (A, E) | F: Unknown;  I: Unknown, Unknown;  T: Unknown;  T: Aerobic, Resistance;  D: 1 - 2 weeks |
| ***Abdominal cancer*** | | | | | | | |
| Pang et al.; Multimodal prehabilitation before major abdominal surgery: A retrospective study | 2021 \| Singapore \| Retrospective cohort \| SS: 591 \| RS: Yes | Supervised and home-based | Preoperatively | Nutritional support; Psychosocial support | Surgical outcomes; Complication rates and LOS; 30 days postoperatively | Psychological; Physical (A) | F: Unknown;  I: Unknown, Unknown;  T: <60 min;  T: Aerobic, Resistance, Balance;  D: Unspecified |
| Waller et al.; Prehabilitation with wearables versus standard of care before major abdominal cancer surgery: a randomised controlled pilot study (trial registration: NCT04047524) | 2022 \| UK \| RCT \| SS: 22 \| RS: No | Home-based | Preoperatively | Nutritional support; Psychosocial support | Physical or functional outcomes; Physical activity assessed via Fitbit and 6MWD; Baseline, before surgery | Psychological; Physical (E) | F: 3x/week;  I: Moderate, Moderate;  T: 60 min;  T: Aerobic, Resistance;  D: 2 weeks |
| Wooten et al.; The Impact of a Multimodal Sport Science-Based Prehabilitation Program on Clinical Outcomes in abdominal Cancer Patients: A Cohort Study | 2022 \| USA \| Cohort study \| SS: 92 \| RS: No | Home-based | Preoperatively | Nutritional support | Surgical outcomes; Complication rates, serious complications, LOS, readmission; 90 days postoperatively | Physical (B); Metabolic; Inflammatory | F: >3x/week;  I: Low, Unknown;  T: 60 min;  T: Blood flow restriction training (aerobic, resistance); D: 4 weeks |
| ***Lung or Upper GI cancer*** | | | | | | | |
| Sheill et al.; Preoperative exercise to improve fitness in patients undergoing complex surgery for cancer of the lung or oesophagus (PRE-HIIT): protocol for a randomized controlled trial | 2020 \| Ireland \| RCT (protocol) \| SS: 78 \| RS: No | Supervised | Preoperatively | Nutritional support | Physical or functional outcomes; Physical fitness assessed using CPET; Diagnoses, Baseline, before surgery | Physical (A) | F: >3x/week;  I: High;  T: <60 min;  T: Interval;  D: 2 weeks |
| ***Intra-cavity cancer*** | | | | | | | |
| Grimmett et al.; The Wessex Fit-4-Cancer Surgery Trial (WesFit): A protocol for a factorial-design, pragmatic randomised-controlled trial investigating the effects of a multi-modal prehabilitation programme in patients undergoing elective major intra-cavity cancer surgery | 2022 \| UK \| RCT (protocol) \| SS: 1560 \| RS: No | Supervised | Preoperatively | Psychosocial support | Surgical outcomes; 1-day reduction; 30 days postoperatively | Psychological; Physical (A) | F: 3x/week;  I: High;  T: <60 min;  T: Interval;  D: Unknown |
| ***Mixed cancer*** | | | | | | | |
| McIsaac et al.; PREHAB study: a protocol for a prospective randomised clinical trial of exercise therapy for people living with frailty having cancer surgery* | 2018 \| Canada \| RCT (protocol) \| SS: 204 \| RS: Yes | Home-based | Preoperatively |  | Physical or functional outcomes; 6MWT; Baseline, 30 days postoperatively | Physical (A, B) | F: 3x/week;  I: Unknown, Unknown;  T: 60 min;  T: Aerobic, Resistance, Stretching;  D: 3 weeks |
| Santa Mina et al.; A Pragmatic Non-Randomized Trial of Prehabilitation Prior to Cancer Surgery: Study Protocol and COVID-19-Related Adaptations | 2021 \| Canada \| Unrandomized controlled study (protocol) \| SS: 150 \| RS: No | Supervised or home-based | Preoperatively | Nutritional support; Psychosocial support; Smoking cessation | Intervention Delivery; Feasibility assessed via program referral rates, enrolment and attrition, intervention adherence and safety, participant satisfaction, and barriers and facilitators to programming.; Intervention period |  | F: 3x/week or more;  I: Low, High, Moderate;  T: 60 min; T: Aerobic, Interval, Resistance;  D: Unknown |
| ***Peritoneal cancer*** | | | | | | | |
| Osseis et al.; Epidural algesia combined with a comprehensive physiotherapy program after Cytoreductive Surgery and HIPEC is associated with enhanced post-operative recovery and reduces intensive care unit stay: A retrospective study of 124 patients | 2016 \| France \| Retrospective cohort \| SS: 124 \| RS: No | Supervised and home-based | Preoperatively |  | Surgical outcomes; Mortality rates, pulmonary complications, LOS; Postoperatively |  | F: >3x/week;  I: Low, Unknown;  T: <60 min;  T: Aerobic, Resistance, Breathing;  D: 4 weeks |

**Study info:** RCT = Randomized controlled trial, SS = Sample size, RS = High-risk patient inclusion criteria;
**Main outcome:** CCI = Charlson Comorbidity Index, 6MWD= Six-minute walk test, LOS = Length of stay, AE = Adverse event, NAC = Neoadjuvant chemotherapy; CPET = Cardiopulmonary exercise test;
**Pathways:** A = Cardiovascular endurance, B = Muscular strength, C = Muscular endurance, D = Flexibility or mobility, E = Body composition;
**FITT and duration:** FITT = Frequency, intensity, time, type, IMT = Inspiratory muscle training, EMT = Expiratory muscle training;
Supervised was defined as >1 supervised session.
*Protocol and results paper were used. The protocol paper is presented.
